# Genome-wide identification and characterization of the chemosensory relative protein genes in *Rhus* gall aphid *Schlechtendalia chinensis*

**DOI:** 10.1186/s12864-023-09322-4

**Published:** 2023-04-28

**Authors:** Hongli He, M. James C. Crabbe, Zhumei Ren

**Affiliations:** 1 School of Life Science, Shanxi University, Taiyuan, 030006 Shanxi China; 2 Wolfson College, Oxford University, Oxford, OX2 6UD UK; 3 Institute of Biomedical and Environmental Science & Technology, University of Bedfordshire, Luton, LU1 3JU UK

**Keywords:** Aphididae, *Schlechtendalia chinensis*, Chemoreception gene family, Genome, Identification

## Abstract

**Background:**

The *Rhus* gall aphid *Schlechtendalia chinensis* specially uses the only species *Rhus chinensis* and certain moss species (Mniaceae) as its primary host plant and secondary host plants, respectively. *Rhus* galls are formed on the primary host by the sucking of aphids, and used in traditional medicine as well as other various areas due to their high tannin contents. Chemoreception is critical for insect behaviors such as host searching, location and identification of mates and reproductive behavior. The process of chemoreception is mediated by a series of protein gene families, including odorant-binding proteins (OBPs), chemosensory proteins (CSPs), olfactory receptors (ORs), gustatory receptors (GRs), ionotropic receptors (IRs), and sensory neuron membrane proteins (SNMPs). However, there have been no reports on the analysis of molecular components related to the chemoreception system of *S. chinensis* at the genome level.

**Results:**

We examined the genes of eight OBPs, nine CSPs, 24 ORs, 16 GRs, 22 IRs, and five SNMPs in the *S. chinensis* genome using homological searches, and these chemosensory genes appeared mostly on chromosome 1. Phylogenetic and gene number analysis revealed that the gene families, e.g., ORs, GRs, CSPs and SNMPs in *S. chinensis*, have experienced major contractions by comparing to *Myzus persicae,* while the two gene families OBPs and IRs had slight expansion. The current results might be related to the broader host range of *M. persicae* versus the specialization of *S. chinensis* on only a host plant. There were 28 gene pairs between genomes of *S. chinensis* and *Acyrthosiphon pisum* in the chemoreceptor gene families by collinear comparison. Ka/Ks ratios (< 1) indicated that the genes of *S. chinensis* were mainly affected by purification selection during evolution. We also found the lower number and expression level of chemoreception genes in *S. chinensis* than in other 11 aphid species, such as ORs, GRs and IRs, which play an important role in host search.

**Conclusion:**

Our study firstly identified the genes of the different chemosensory protein gene families in the *S. chinensis* genome, and analyzed their general features and expression profile, demonstrating the importance of chemoreception in the aphid and providing new information for further functional research.

**Supplementary Information:**

The online version contains supplementary material available at 10.1186/s12864-023-09322-4.

## Background

The *Rhus* gall (or sumacgall) aphids switch host plants between the primary host plants *Rhus* (Anacardiaceae) species and the secondary hosts certain mosses to complete their life cycles, and form galls on their primary host plants [1–3]. The galls, often used as the Chinese medicines, are rich in tannins and economically important in Asia because they have medicinal properties and represent sources of industrial tannin [4, 5]. This aphid group belongs to the subtribe Melaphidina in tribe Fordini (Aphididae: Eriosomatinae) [6–8], and includes six genera and 13 species [3, 9], among which *S. chinensis* is the most common and wide-spread species with *R. chinensis* as its unique primary host plant and Mniaceae species as its secondary hosts, as well as having a life cycle including both sexual and asexual reproduction stages [10, 11].

The chemosensory system is critical for insects to detect and locate suitable host plants [12]. It has been demonstrated that this behavior is mediated by several protein gene families, such as odorant-binding proteins and chemosensory proteins (OBPs and CSPs) gene families, and olfactory receptors, gustatory receptors and ionotropic receptors (ORs, GRs and IRs) gene families, and sensory neuron membrane proteins (SNMPs) gene families [13–15].

OBPs are small, globular and water-soluble proteins that play an important role in the first step of olfactory recognition [16, 17]. The hallmark of the protein family is the six conserved cysteines which contain three paired disulfide bridges [18]. Based on the number of cysteine residues they contain, OBPs are now classified into four types, i.e., “Classic”, “Minus-C”, “Plus-C”, and “Atypical” [19]. Since the first OBP was identified in *Antheraea polyphemus* [20], a large number of OBP genes have been identified from different insect species [19, 21]. CSPs are small, soluble, acidic proteins composed of five α helices and four conserved cysteines with two disulfide bridges [22]. Like OBPs, the CSPs are also regarded as the first step for the transportation of odorants in chemosensory recognition and widely identified in almost all insect groups [23]. The first CSP member called P10 was identified in the American cockroach *Periplaneta americana* [24], and then a second was found in *Drosophila* antennae named OS-D (olfactory segment D) or A-10 [25].

ORs are members of the G-protein-coupled receptor family with seven transmembrane domains, composed of 300 to 500 amino acids. The ORs, such as the olfactory receptor co-receptor (Orco) and conventional ligand-binding odorant receptors, play key roles in olfactory behavior [26]. The ORs not only recognize odor molecules alone but can form heteromeric complexes with Orco. The sequence of common ORs is highly differentiated among different insects with low homology, generally 20%. Orco is highly conserved among different insects and the homology among different species can be more than 70% [27]. The OR family originated from the GR family at the base of the insects [28]. The GR family is far older than the OR family in animals and consists of several major subfamilies [29]. GR genes were initially screened in *Drosophila melanogaster* [30], which consists of seven hydrophobic transmembrane (TM) domains with approximately 300–500 amino acids. They are divided into four major subfamilies regarding their active ligands: fructose, sugars except fructose, carbon dioxide (CO_2_) and bitter receptors [31]. GRs, similar to ORs, may be ligand gated ion channels, most of which are divergent and have low sequence identity between insect species [32]. The IR gene family is a variant of the ionotropic glutamate receptor (iGluR), which was initially found in *D. melanogaster* using bioinformatic techniques [33]. According to the amino acid sequence and gene expression pattern, IRs are generally divided into three subfamilies: olfactory, differentiated, and co-receptor IRs [34]. IRs need to be co-expressed with IR co-receptors to function. At present, four IR co-receptors have been found, namely IR8a, IR25a, IR76b and IR93a, which are relatively conservative among insect species [35].

SNMPs are the transmembrane domain-containing proteins and belong to a large gene family of CD36 receptors [36]. They are composed of 520 amino acids and divided into two subfamilies: SNMP1 and SNMP2. The homology of SNMP1 and SNMP2 within the same species is relatively low, only 20-30% [37]. Among different species, the homology of SNMP2 is higher than SNMP1. SNMP1 found in *D. melanogaster* is the first insect SNMP gene, that was functionally characterized to be essential for its sex pheromone detection [38].

The chemoreception genes play a decisive role in the host search of insects, and the *Rhus* gall aphid *S. chinensis* is so specific to choose either the primary *Rhus* host or secondary moss hosts. However, there is no report on the chemoreception genes in the *Rhus* gall aphid *S. chinensis*. Here, we used the third-generation sequencing technology to obtain the whole genome of *S. chinensis* at the chromosome level and performed the comprehensive analysis of six gene families in the *S. chinensis* genome. In detail, we conducted systematic identification and molecular characterization, which included the member identification of gene family, collinear analysis, chromosomal location, evolutionary selection pressure (Ka/Ks analysis), and gene expression analysis. We highlighted the characters of the key genes in the chemoreception protein gene families to further investigate the mechanism of the *S. chinensis –* host plant adaptive interactions for future functional research.

## Results

### Characteristics of chemoreception genes in *S. chinensis*

We identified the genes and analyzed their characteristics in the six chemoreception gene families by homological search in the *S. chinensis* genome, where we finally obtained eight genes in OBPs, nine genes in CSPs, 24 genes in ORs, 16 genes in GRs, 22 genes in IRs and five genes in SNMPs. To better understand the evolutionary relationships and structural components of chemoreception genes in *S. chinensis*, we analyzed the conserved motifs, domain, exon–intron gene structures and phylogenetic relationship based on genome sequences and protein sequences.

**Fig. 1:**
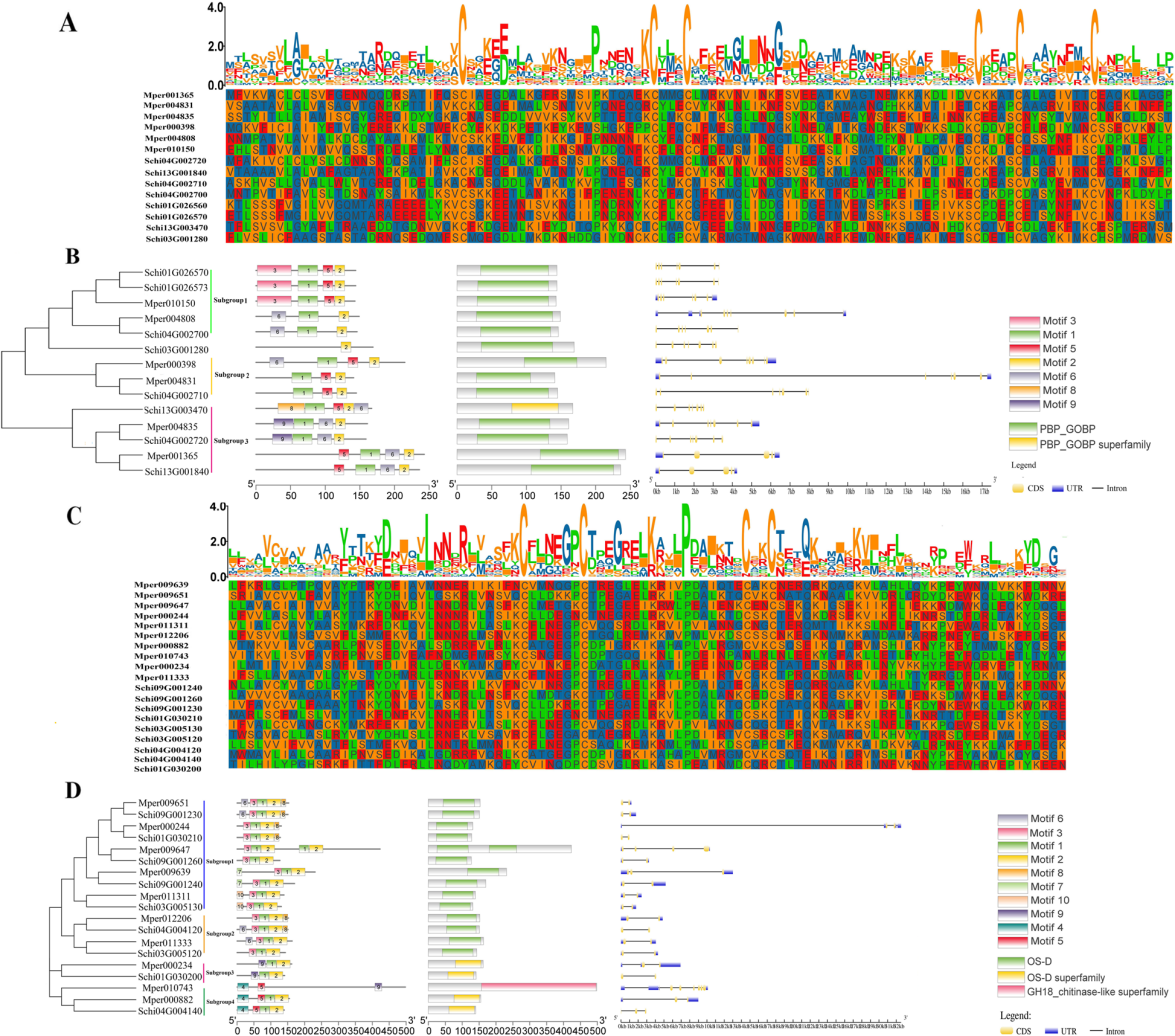
The general characteristics of OBPs and CSPs gene sequences in *Schlechtendalia chinensis.* Gene sequence alignment of OBPs (**A**) and CSPs (**C**), and phylogenetic relationships, conserved motifs, domains and gene structures analysis of OBP (**B**) and CSP (**D**) gene family in *S. chinensis* and *M. persicae*

The protein sequence analysis on the OBP genes of *S. chinensis* (Fig. 1A) showed that four of eight OBPs belonged to the classical OBP subclass with the typical six conserved cysteine residues, and the other four OBPs belonged to the Plus-C subclass. The phylogenetic tree of the *S. chinensis* and *M. persicae* OBPs divided the sequences into three subgroups (Fig. 1B). The motif of sequence from same subgroup was not conservative, which might arise from the low conservation between the gene sequences of OBPs. The conserved domain of all the gene sequences included PBP_GOBP. The numbers of exons ranged from three to eight from predictions of the gene structure. A total of seven members exhibited 5′ and 3′ UTRs, while seven members presented no UTR. All the gene sequences of CSPs in the *S. chinensis* genome contained four highly conserved cysteine residues, which are characteristic of typical insect CSPs (Fig. 1C). The phylogenetic tree constructed by CSPs gene sequences from the *S. chinensis* and *M. persicae* genomes suggested that all the sequences were divided into four subgroups (Fig. 1D). Subgroup 1 and subgroup 2 were relatively conservative and included motif 3-1-2. Subgroup 3 and Subgroup 4 included motif 9-1-2 and motif 4-5-1-2, respectively. The conserved domain contained OS-D and GH18_chitinase. The protein gene sequences were less conserved, which might be related to their relative relationship and feeding habits. The numbers of exons ranged from two to five from the predictions of the gene structure. Gene length varied among the CSPs, among which genes with a length less than 10 kb accounted for the majority (88%), while genes longer than 10 kb accounted for a small portion. Major members exhibited 5′ and 3′ UTRs, while four members presented no UTR.

**Fig. 2:**
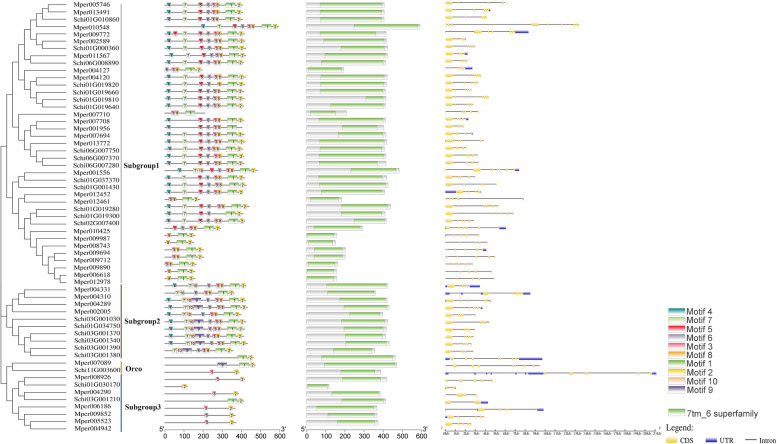
Phylogenetic relationships, conserved motifs, domains and gene structures of the OR gene family in *S. chinensis* and *M. persicae*

**Fig. 3:**
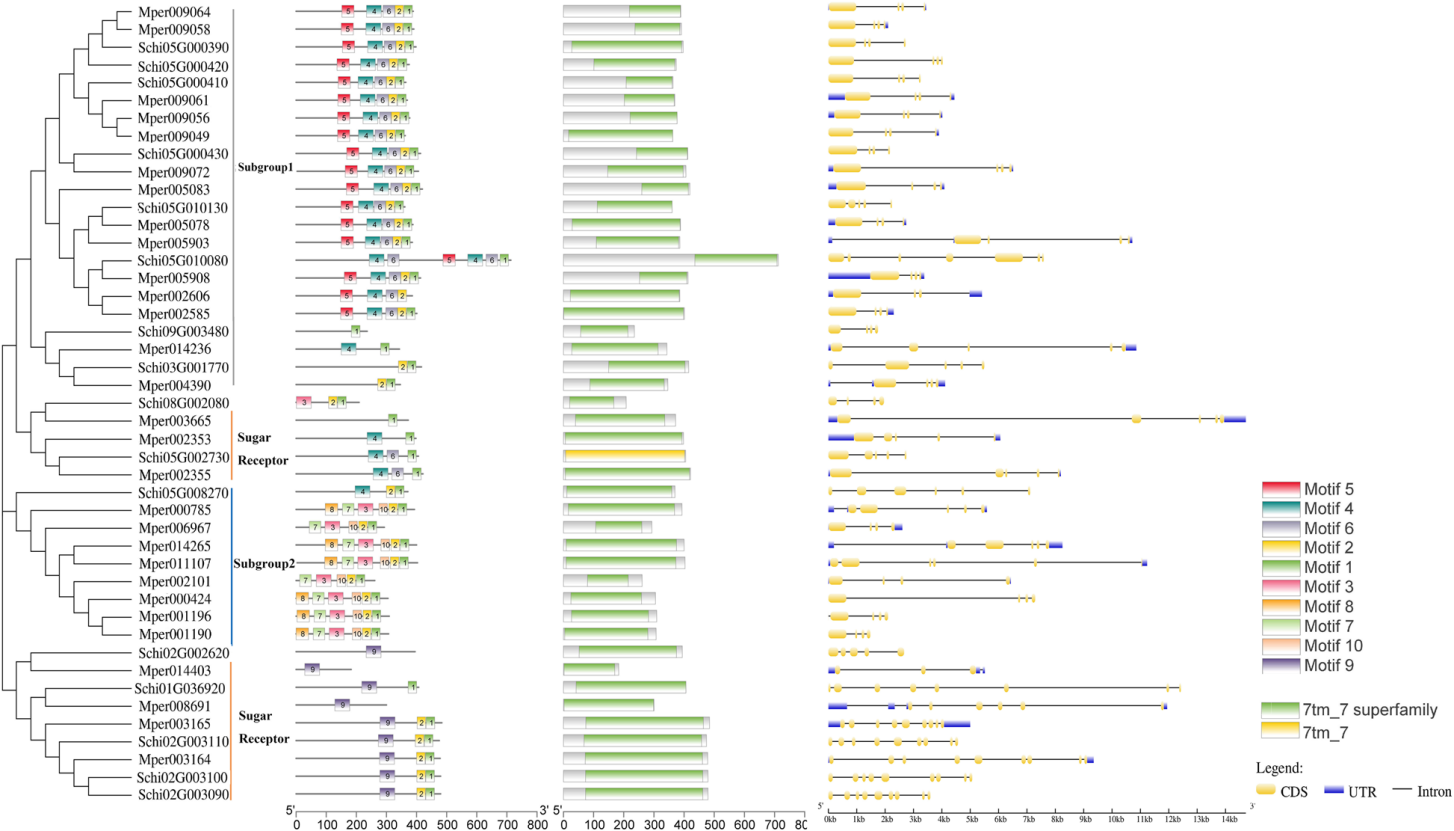
Phylogenetic relationships, conserved motifs, domains and gene structures of the GR gene family in *S. chinensis* and *M. persicae*

In the *S. chinensis* genome, the OR family consists of the single highly conserved Odorant receptor co-receptor (Orco) and 23 “specific” ORs, each of which is thought to pair with Orco to form a functional olfactory receptor tetramer. The phylogenetic tree of the OR protein gene sequences in the *S. chinensis* and *M. persicae* genomes were distributed in three subgroups (Fig. 2). The members of subgroup 1 and subgroup 2 were conservative with motif order 4-7-5-6-3-8-1-2 and 4-7-10-9-6-3-8-1-2, respectively. The conserved domain of protein gene sequences contained 7tm_6. The numbers of exons ranged from two to nine from the predictions of the gene structure. Gene length varied among the ORs, among which genes with a length less than 10 kb accounted for the majority, while few genes were longer than 10 kb. Major members presented no UTR, and 12 members exhibited 5′ and 3′ UTRs. The phylogenetic tree constructed by the GR’ protein sequences in the *S. chinensis* and *M. persicae* genomes were distributed in four subgroups (Fig. 3), and seven GRs genes of *S. chinensis* belonged to sugar receptors. There are ten conservative motifs in the GRs gene sequences of *S. chinensis* and *M. persicae*. The motif orders of subgroup 1 and 2 were motif 5-4-6-2-1 and motif 8-7-3-10-2-1, respectively. The frequency of motif 1 was the highest, which existed in all gene sequences except for Schi02G002620. In the gene family, some motifs were found only in a subfamily. For example, motif 9 just existed in sugar receptor subgroup genes. The conservative domain was similar to ORs, e.g., 7tm_7, which might be related to the origin of ORs from GRs. The structural analysis suggested that the longest gene was 14 kb in the GRs, and most genes existed four or five exons accounted for the majority (77.7%). However, there may be a small subset of genes containing 9 or 10 exons, e.g., Schi02G003090 and Schi02G003100.

**Fig. 4:**
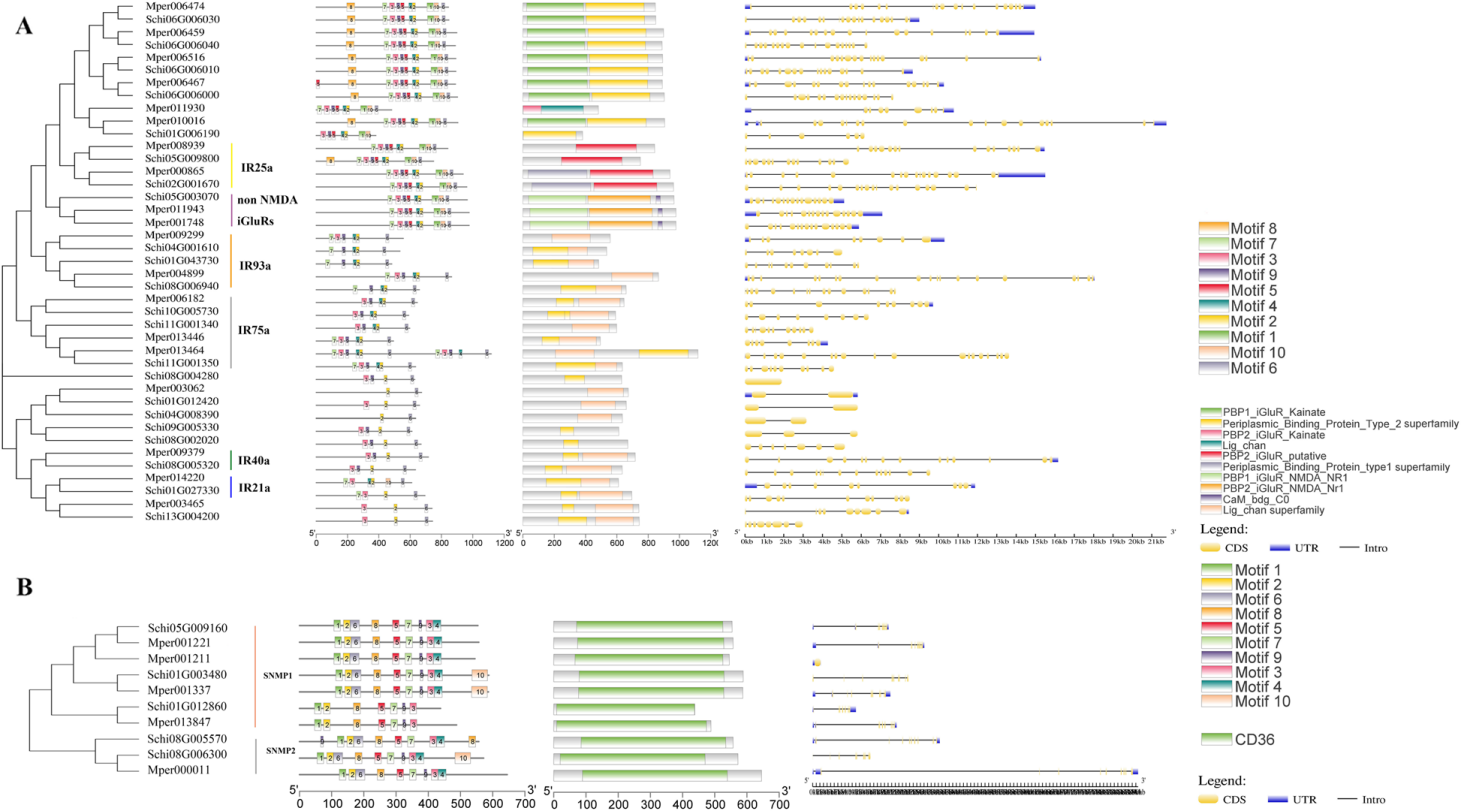
Phylogenetic relationships, conserved motifs, domains and gene structures of the IR (**A**) and SNMP (**B**) gene family in *S. chinensis* and *M. persicae*

The 22 IRs in the *S. chinensis* genome included IR25a, IR21a, IR40a, IR93a, IR75a and iGluRs subfamily. Two members (Schi05G009800 and Schi02G001670) from IR25a with subsets of the other IRs are the most conserved members of the gene family IRs (Fig. 4A). Schi01G027330 and Schi08G005320 belonged to IR21a and IR40a, respectively, and three members belonged to IR93a which were secondary most conserved genes. In addition, one gene and three genes belonged to non NMDA iGluRs and IR75a, genes of which in are involved in perception of various acids. Phylogenetic results from the *S. chinensis* and *M. persicae* IRs gene sequences showed that all the sequences were divided into five subgroups and the motif of each part was conservative. All sequences had the motif order 3-2-6, while the complete motif order was motif 8-7-3-9-5-4-2-1-10-6. The conservative domain included PBP1_iGluR_Kainata and PBP1_iGluR_NMDA. The numbers of exons ranged from two to 19 from predictions of the gene structure, and the longest gene was 21 kb in the IRs. Most genes included more than 10 exons accounted for 68.2%. Thirty-four percent of members exhibited 5′ and 3′ UTRs, while 14.6% of members presented 5′ or 3′ UTR and 20% members had no UTR. Just one member from *M. persicae* had four UTRs*.*

The phylogenetic trees of SNMPs in *S. chinensis* and *M. persicae* divided the sequences into two subgroups: SNMP1 and SNMP2 (Fig. 4B). Three genes of *S. chinensis* belonged to SNMP1 and the sequences had the same motif order 1-2-8-5-7-9-3, which were as a co-receptor. Two genes belonged to SNMP2 with the same motif order 1-2-6-8-5-7-9-3-4. The conservative domain belonged to a large gene family of CD36 receptors. The numbers of exons was nine or ten, except for Mper001211 with one from predictions of the gene structure. Just two members had no UTR, and others exhibited 5′ and 3′ UTRs.

### Chromosomal location and collinearity of chemoreception genes in* S. chinensis*

**Fig. 5:**
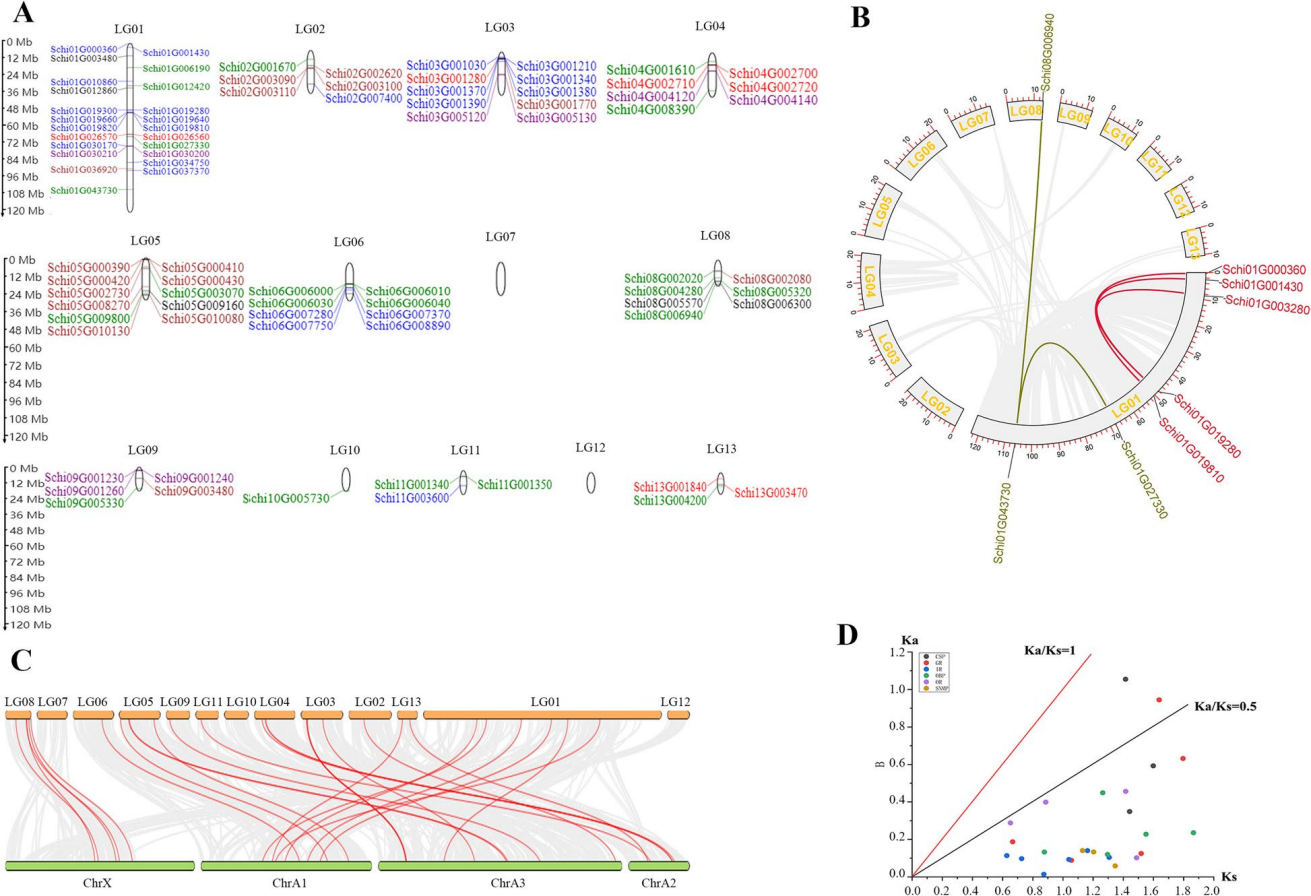
Location and collinearity analysis of all chemoreception genes in *S. chinensis*. **A** Scaffold location and gene tandem. Green represent IRs; Blue represent ORs; brown represent GRs; black represent SNMPs; Red represent OBPs; purple represent CSPs **B** Chromosomal location and collinearity. Grey boxes represent chromosomes. Lighted lines connect chemoreception gene duplication. **C** Synteny on gene families of *S. chinensis* and *Acyrthosiphon pisum*. **D** Ka/Ks ratios of chemoreception genes of *S. chinensis*

The location and collinearity analysis of all 84 chemoreception genes showed that they were unevenly distributed on chromosomes 1-13, except for chromosome 7 and 12 (Fig. 5A).

Chromosome 1 had the most members of chemoreception genes with 23 genes, among which there were 12 genes in ORs, four genes in IRs, two genes in SNMPs, OBPs and CSPs, respectively, one gene in GRs. Chromosome 10 had the fewest chemoreception genes with only one member. The distribution of the genes on chromosomes showed no bias to the 5′ or 3′ ends, which may be related to their function. There were five pairs of genes in the chemoreception genes which had collinearity, i.e., Schi01G027330 and Schi01G043730, Schi01G043730 and Schi08G006940 from the IR gene family; Schi01G000360 and Schi01G019810, Schi01G001430 and Schi01G019280, Schi01G019280 and Schi01G003280 from the OR gene family. There was no gene tandem replication on chromosome 1 (Fig. 5B). The collinear comparison map of the chemoreception gene family between *S. chinensis* and *A. pisum* was established by MC Scan X (Fig. 5C). There were 28 pairs of collinearity (homologous gene pairs) in *S. chinensis* and *A. pisum* genome, including three in CSPs and SNMPs, five in GRs, OBPs and ORs, and seven in IRs. There were more homologous gene pairs for IRs between the *S. chinensis* and *A. pisum* genome, which may be related to the large number of the gene families.

The Ka/Ks ratio has been used for genomic analysis of gene families, which can provide insights into selective evolutionary pressures that act on genes. To better understand whether chemoreception genes in *S. chinensis* and *A. pisum* were subjected to different evolutionary constraints, the pairwise Ka/Ks was calculated for each ortholog group (Fig. 5D). The Ka/Ks analysis of 28 pairs of homologous genes existing in *S. chinensis* and *A. pisum* was carried out. The ratios of Ka/Ks between gene pairs were all < 1, which indicated that negative selection (purification selection) drove chemoreception gene family evolution as the primary force in two species. However, the Ka/Ks ratios of two genes from CSPs and GRs were much higher than others, which indicated that they had undergone positive selective pressure.

### Evolution of chemoreception genes in *S. chinensis*

**Fig. 6:**
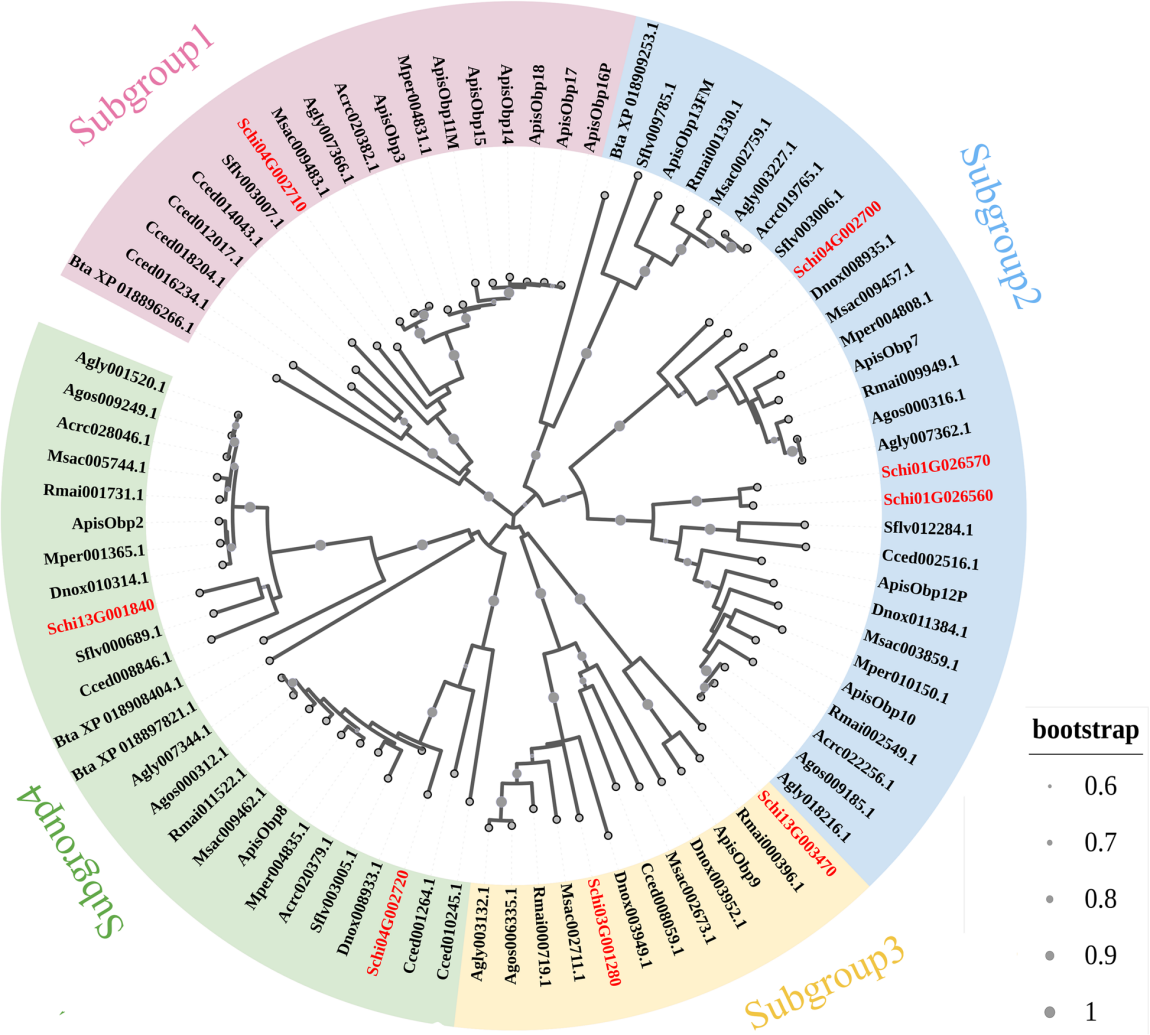
Neighbor-joining tree of OBPs gene sequences in *S. chinensis* and other Hemiptera species. The genes of *S. chinensis* are highlighted in red shadow. All gene names are the abbreviation of the species name plus the gene serial number, and the gene serial number could be found in Insect Base 2.0 (http://v2.insect-genome.com/). Cced, *Cinara cedri*; Mper*, M. persicae*; Apis, *A. pisum*; Agly, *Aphis glycines*; Agos*, Aphis gossypii*; Dnox*, Diuraphis noxia*; Rmai*, Rhopalosiphum maidis*; Msac, *Melanaphis sacchari*; Acra, *Aphis craccivora*; Sflv, *Sipha flava*; Bta, *Bemisia tabaci*

**Fig. 7:**
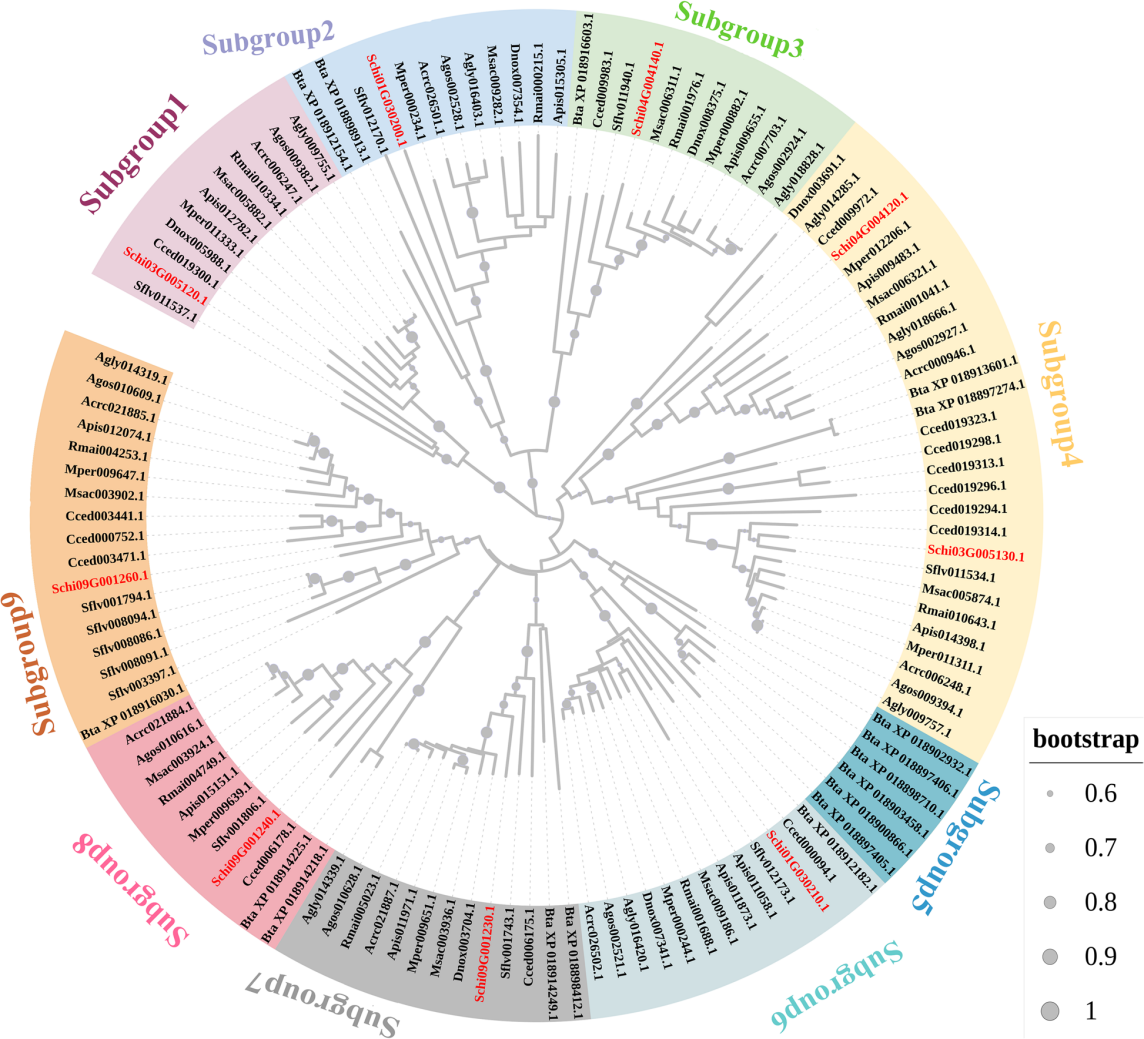
Neighbor-joining tree of CSPs gene sequences of *S. chinensis* and other Hemiptera species. The gene of *S. chinensis* is highlighted in red shadow. Gene names are same as Fig. 6

For estimating the evolutionary relationship among chemoreception genes of *S. chinensis*, the six chemoreception protein gene families from 12 Hemiptera species were used to construct the NJ phylogenetic tree, respectively. Among the investigated species, the gene number of OBPs in the *A. pisum* genome was the most with 10 OBPs, followed by *C. cedri* with nine OBPs*.* The other species have less than eight OBPs (Table 1). The phylogenetic analysis of the OBP genes in the 12 species included in this study showed that the genes were clustered into four clades, and the eight OBP genes of *S. chinensis* occurred in four clades (Fig. 6). The number of CSP genes in *S. chinensis* (nine members) was the same as in *R. maidis*, *M. sacchari*, *A. craccivora* and *A. gossypii*, while less than the other species, which have more than 10 CSPs; *B. tabaci* had the most CSP genes with 17 members. The CSPs were phylogenetically clustered into nine clades, while CSPs in *S. chinensis* occurred in eight clades lacking a gene of subgroup 5 (Fig. 7). It indicated that *S. chinensis* might loss the function of a homologous gene from subgroup 5. We identified 24 ORs in *S. chinensis*. Several OR candidate genes were identified in each of the 12 species studied, while the species *A. pisum*, *M. persicae*, *R. maidis* and *A. gossypii* have over 24 ORs. The phylogenetic tree showed that the OR genes in *S. chinensis* were clustered into five clades (Fig. S1), which was consistent with the phylogenetic tree division of 12 species. A total of 16 GR genes were identified in *S. chinensis*, which were divided into seven clades (Fig. S2). We found less than 32 GRs (*A. gossypii*) in each of the 12 species studied. The 22 IR genes of *S. chinensis* were dispersed in 10 clades. The 12 Hemiptera species generally had 18 to 24 IRs, except *B. tabaci* with 30 IRs (Fig. S3). Similarly, the candidate SNMP genes were clustered into eight subgroups. The SNMPs of *S. chinensis* occurred in all subgroups except subgroup 2, 4 and 7. The numbers of candidate SNMP genes identified in the genomes of the 12 species ranged from five to nine, with 21 in *B. tabaci* (Fig. S4).

**Table 1:** Chemoreception gene numbers of 11 Aphididae and one Aleyrodidae species

Family	Species	GRs	ORs	OBPs	IRs	CSPs	SNMPs	Total
Aleyrodidae	*Bemisia tabaci*	16	2	5	30	17	21	91
Aphididae	*Sipha flava*	15	19	6	19	12	8	79
	*Cinara cedri*	23	22	9	22	15	9	170
	*Schlechtendalia chinensis*	16	24	8	22	9	5	84
	*Acyrthosiphon pisum*	26	36	10	24	10	8	114
	*Diuraphis noxia*	12	8	6	21	11	8	198
	*Myzus persicae*	29	37	6	19	10	8	109
	*Rhopalosiphum maidis*	29	33	8	21	9	9	109
	*Melanaphis sacchari*	24	23	8	18	9	8	218
	*Aphis craccivora*	22	20	6	24	9	8	89
	*Aphis glycines*	25	22	8	21	10	9	95
	*Aphis gossypii*	32	30	6	18	9	9	184

### Expression profiles of chemoreception genes in* S. chinensis*

We examined 17 chemoreception genes in the transcriptome data of *S. chinensis*, among which there were six genes in CSPs, two genes in GRs, four genes in IRs, one gene in OBPs and ORs, three genes in SNMPs, respectively. The expression of Schi01G030200 and Schi09G001260 from CSPs was the highest with 285.82 FPKM, while Schi02G001670 in IRs was the least (Table 2). The genes with the more number of copies, for example ORs, GRs, IRs, had lower expression level, which might mainly result from the host specificity of the *Rhus* gall aphid *S. chinensis* for these genes play an important role in looking for hosts.

**Table 2:** Expression profiles of chemoreception genes in *Schlechtendalia chinensis* at the stage of alate migrants

Gene family	Gene ID	Transcriptome ID	Ren_IA4601	Ren_IA4603	Ren_IA462
CSPs	Schi01G030200	TRINITY_DN483_c0_g1	251.79	366.84	238.82
	Schi03G005120	TRINITY_DN1981_c0_g2	47.62	2.42	12.99
	Schi04G004120	TRINITY_DN20590_c0_g1	4.19	1.1	0.4
	Schi04G004140	TRINITY_DN795_c2_g1	57.34	91.5	104.1
	Schi09G001240	TRINITY_DN4954_c0_g1	25.77	14.98	15.35
	Schi09G001260	TRINITY_DN3437_c0_g1	199.37	161.65	73.86
GRs	Schi02G002620	TRINITY_DN5285_c0_g1	14.72	22.67	8.8
	Schi05G002730	TRINITY_DN16137_c0_g1	1.62	1.05	1.72
IRs	Schi02G001670	TRINITY_DN22358_c0_g1	0.82	0.52	1.39
	Schi05G003070	TRINITY_DN3565_c0_g1	11.32	21.28	10.89
	Schi05G009800	TRINITY_DN15399_c0_g1	0.91	0.93	0.85
	Schi06G006010	TRINITY_DN2800_c0_g1	24.94	36.05	17.29
OBPs	Schi01G026570	TRINITY_DN6444_c0_g1	8.46	4.03	9.54
SNMPs	Schi01G012860	TRINITY_DN6299_c0_g1	3.24	5.71	2.97
	Schi08G006300	TRINITY_DN635_c0_g1	100.93	45.98	104.4
	Schi01G003480	TRINITY_DN2496_c0_g1	9.9	10.51	8.43
ORs	Schi11G003600	TRINITY_DN21115_c0_g1	2.49	0.34	1.71

the unit of gene expression level is Fragments Per Kilobase of exon model per Million mapped fragments (FPKM)

## Discussion

The species *S. chinensis* is predominant in the *Rhus* gall aphids, and is widely distributed in East Asia, mainly in China, and economically valuable because it lives on its primary host plant *Rhus chinensis* in the family Anacardiaceae to form galls with high tannins [5]. In addition, this aphid species used only *R. chinensis* as its unique primary host plant and several moss species (Mniaceae) as its secondary host plants, and they have evolved as an obligate mutualism relationship [39]. Chemoreception relative genes play important roles in the host finding process [12]. In this study, we identified the chemoreception gene family of *S. chinensis* at the whole genome level, and analyzed its basic characteristics including motif, conserve domain and gene structure. Moreover, the collinearity, evolution and expansion/contraction of chemoreception revealed evolutionary relationships of chemoreception relative genes in aphids with different feeding habits.

We identified 84 chemoreception genes in *S. chinensis*, which was the least by comparison to other aphids including *A. pisum* (114), *M. persicae* (109), *A. gossypii* (184) and *D. noxia* (198). In the subfamily Aphidinae, *A. pisum*, *M. persicae* and *D. noxia* belong to tribe Macrosiphini, while the other species belong to the tribe Aphidini [40]. *Acyrthosiphon pisum* was the first aphid species that had its genome completely sequenced and there are many gene replication events [41]. *A. gossypii* feeds on widely feed diverse crops in the species of the families Malvaceae, Rutaceae, and Cucurbitaceae [42]. *D. noxia* feed on the members of *Gramineae* family. Furthermore, *A. pisum* and *D. noxia* are oligophagous, while *A. gossypii* and *M. persicae* are polyphagous. Insects utilize their senses of taste and smell to determine whether to feed on certain plants [43]. Thus, the number of chemoreception genes is closely related to eating habits and characteristics and types of host plant [44]. *S. chinensis* feeds on only limited host plant and has fewer chemoreception genes than other aphids. In addition, OBPs and CSPs are regarded as the first step of host recognition, and the number of OBPs and CSPs were significantly less than GRs, ORs and IRs in *S. chinensis*, which suggested that *S. chinensis* has poor host recognition. This maybe also related to its special and oligophagous host plants.

We performed a characteristic analysis of six chemoreception gene families in *S. chinensis* and *M. persicae*. The chemoreception genes in the same groups had different motif patterns, which might be the reason for the differences in their physiological functions. The protein sequence of OBPs showed less conservation in *S. chinensis*, which was consistent with the previous study that the protein sequences of OBPs had low similarity and were highly differentiated between species and within species [45]. Compared with OBPs, CSPs are relatively conserved in evolution, and have high sequence similarity among different species, including *S. chinensis*. Both of them are key proteins involved in sensing chemical information, and usually contain multiple relatively conserved cysteine (Cys) [46]. The conserved domain was PBP_GOBP and OS_D, exhibiting the typical characteristics of insect OBP and CSP. The PBP_GOBP and OS_D domain of proteins are involved in general odor-binding activities, which have the function of recognizing, binding and transporting chemical substances such as odor molecular pheromones in the process of host search [47]. In *S. chinensis*, the gene structure of some members within the same subfamily showed similar intron/exon structure and intron phases. The conserved domains of GRs and ORs had a similarity which both belonged to the 7tm superfamily. This may be related to the fact that OR evolved from GR [28]. ORs and GRs of the *S. chinensis and M. persicae* genomes in the same class had similar motif patterns and gene structure. The ORs of two aphids had Orco which has the same function in different insects and plays a key role in the process of insect olfactory recognition. GRs in *S. chinensis* only had the sugar receptor, which are partially co-expressed in a single GRN of each taste sensillum and primarily responsible for the insect’s ability to accurately find sugars and avoid toxic substances [48]. The IR family is evolutionarily independent of the OR/GR gene families, which together form the insect chemoreceptor superfamily [36]. The IRs in *S. chinensis* included IR25a, IR75a and IR93a, which belonged to co-receptor IRs and were conserved between insect species [49]. The protein sequences of IRs in the same clade shared similar motifs and same conserve domain, but the gene structure had some differences. High variation in the sequence structure revealed that IRs family members have acquired changes in their genome during evolution events that affected their functions [50]. The ORs, GRs, and IRs deliver chemical pheromones or environmental odors to the chemoreceptors of sensory neurons in the process of host search [51]. The gene number of SNMPs was fewer than other chemoreception gene families and the gene structure variation of the homologous SNMP1 and SNMP2 in the same species was low [52]. SNMPs dendrite membranes that assist ORs in the process of sex pheromone recognition in *S. chinensis*. The gene family in any species have classic domains, indicating a relatively conservative evolutionary pattern to ensure functional stability. However, the structural domains, motifs and gene structure of the members of the same subfamily show a more or less conserved pattern, implying differentiation of function and also representing different selective pressures [53].

The phylogenetic results showed that chemoreception genes of *S. chinensis* were distributed closely to *S. flava*, *D. noxia* and *C. cedri*. This indicated that gene family clustering was not necessarily based on species affinity, but clustered according to similar functions. The number of genes differ among species of Hemiptera, which is related to the complexity of the chemoreceptor genes in these species. In Hemiptera, the number of OBP genes varies greatly among species. We found an expansion of the OBP family in *A. pisum*, relative to other species. In addition, *B. tabaci* in Aleyrodidae had a contraction of OBP family. The number of CSP genes showed differentiation among species. Among all species, *S. chinensis* had the lowest number of the three kinds of soluble protein genes, which may be attributed to its specificity to its plant host. Each of the 12 Hemiptera species have a large OR and GR family. Tandem duplication has been extensively found in the OR family, and the expansion of the OR family is usually accompanied with contraction of the GR family [54]. In addition, the numbers of SNMPs show difference among Hemiptera species. *Bemisia tabaci* showed marked expansion. *S. chinensis* lacked genes in three subgroups, which suggested a partial lack of function. Among all the 12 species, *M. sacchari* has the most abundant chemoreceptor genes. The difference in the number of gene family members may be due to gene duplication or loss in the process of gene evolution. Gene duplication and loss were the main evolutionary driving forces for the expansion or contraction, and duplicated genes could lead to gene redundancy [55].

The collinearity analysis showed that chemoreception gene family of *S. chinensis* has experienced duplication events. It was reported that gene duplications were critical for the evolution of new genes and novel functions, which were the major forces for driving gene family expansion [56]. Notably, the values of Ka/Ks for all gene pairs in *S. chinensis* suggested that they were under strong negative selection pressures. A similar evolutionary pattern was observed in the *D. melanogaster* genome, in which purifying selection was the main selection pressure driving the diversities of ORs, GRs and OBPs [57]. Additionally, all Ka/Ks values of the chemoreception gene family were further away from 1 with IRs lower than the values of other gene family, suggesting that they experienced stronger selective pressures and needed shorter genes to duplicate easily that took less time.

## Conclusions

In the present study, we identified chemoreception gene families including eight OBPs, 16 GRs, 24 ORs, 22 IRs, nine CSPs and five SNMPs in the *S. chinensis* genome. Gene structure and protein motif analysis suggested that chemoreception genes in different families were conservative in *S. chinensis*. Synteny analysis showed that many chemoreception genes demonstrated a favorable collinearity within *A. pisum* and were undergoing a purifying selection, and several pairs of chemoreception genes of *S. chinensis* experienced duplication events. The gene family expansion/contraction and phylogenetic analysis revealed that the chemoreception gene families significantly contracted during the evolution of *S. chinensis*, and *A. pisum* had most chemoreception genes. Furthermore, transcriptome data showed that only a few chemoreception genes were expressed in *S. chinensis*. All in all, our study firstly identified the chemoreception genes of the different gene families in the *S. chinensis* genome, and analyzed their general features and expression in detail, and highlighted the characters of the chemoreception genes in the *S. chinensis*-host adaptive interactions, which will afford important basic information for the further functional studies.

## Methods

### Sample information

The mature *Rhus* galls formed by the species *Schlechtendalia chinensis* were collected from its host plant *Rhus chinensis* in the *Rhus* gall breeding base in Wufeng county (30°19′ N, 110°67′ E, 329 m above sea level), Hubei Province, China. The base has cultivated *Rhus* gall for more than twenty years, and specially the botanist Jun Wen from the Smithsonian Institution, US, visited the base in 2019 and confirmed the host plant *Rhus chinensis*. About 30 live aphid individuals from one gall were used to extract the total genome DNA for genomic sequencing by the third-Generation high throughput technology, which was performed with the sequencing depth of 60 × through the PacBio platform of Biomarker Technologies Corporation (Beijing, China). Fundatrigeniae with wings and without wings in a gall were from the same clone to be treated as one sample. We also collected alate migrants from three mature galls, and the aphids in this stage will look for and fly to winter hosts as soon as they are out from the natural open gall. Total mRNA of *S. chinensis* individuals from these three mature galls were extracted and sequenced using an Illumina HiSeq 2500 device in a 2 × 150 paired-end format. All the clean reads were used together for assembling for transcription sequencing to characterize the chemoreception gene expression pattern in *S. chinensis* genome. There are thousands of clonal individuals from one fundatrix in one gall, and some of the aphid individuals were used for sequencing, and the others were stored as the specimen, which are deposited in the herbarium at School of Life Science in Shanxi University, China, with the voucher number Ren_IA4601, Ren_IA4602, Ren_IA4603, Ren_IA4621.

### Identification of chemoreception gene families in *S. chinensis*

All protein-coding sequences of the families OBPs, GRs, ORs, IRs in the *S. chinensis* genome were searched against the protein database of another aphid species *Acyrthosiphon pisum* [58] by applying BLASTP (e-value = 1 × 10^−5^ and identity >  = 40). The protein-coding gene sequences of CSPs and SNMPs in *S. chinensis* were obtained by searching in the annotation table, which was obtained by integrating three approaches, namely de novo prediction, homology search, and transcript-based assembly, by using the key words of chemoreception genes. All sequences were determined by the NCBI Conserved Domain Database (NCBI-CDD) (e-value = 1 × 10^−3^), and the sequences that lacked conservative structures were discarded [59]. All sequences verified by the two methods were considered as potential genes.

### Protein motif and structure of chemoreception genes

The protein sequences of the species *Myzus persicae* were downloaded from the Insect BASE website (http://v2.insect-genome.com/). The chemoreception protein sequences of *M. persicae* were obtained by blast with homologous gene of *A. pisum* (e-value = 1 × 10^−5^ and identity >  = 40). A neighbor-joining (NJ) tree was established using MEGA-X with 1000 replicates of bootstrap [60]. The conserved structure alignment of chemoreception protein sequences was performed by TBtools. Conserved motifs were identified via Motif-based sequence tools (MEME, http://meme-suite.org/) with the number of motifs as 10 [61], and the conserved domain was analyzed by the Conserved Domain Database (NCBI-CDD) (e-value = 1 × 10 − 3). The exon and intron structures were displayed in all gene sequences using the Gene Structure Display Server (GSDS) (http://gsds.cbi.pku.edu.cn/). TBtools was used to visualize and merge the results of basic characteristics which contained the phylogenetic tree, motif pattern, domain and gene structure [62].

### Chromosomal locations, synteny analysis and Ka/Ks calculation

To understand the distributions of chemoreception genes on chromosomes, positional information was extracted from the GFF3 profile of *S. chinensis* and displayed on the 13 chromosomes via MG2C (http://mg2c.iask.in/mg2c_v2.1/) [63]. MCScanX was leveraged to detect the collinearity and duplication events in the intra- and inter- specific relationship of the chemoreception genes family of *S. chinensis* and *A. pisum* genomes [64]. The ratios of synonymous (Ks) and non-synonymous (Ka) nucleotide substitutions (Ka/Ks) of homologous gene pairs were also calculated via the Simple Ka/Ks Calculator of TBtools [62], in which Ka/Ks < 1 indicated purifying selection.

### Phylogenetic analysis of chemoreception genes

In order to analyze the phylogenetic relationship of chemoreception protein gene families in Hemiptera, 12 species were selected, including 11 species in Aphidinae, i.e., *Cinara cedri*, *M. persicae*, *A. pisum*, *Rhopalosiphum padi*, *Aphis glycines*, *Aphis gossypii*, *Diuraphis noxia, Rhopalosiphum maidis*, *Melanaphis sacchari*, *Aphis craccivora*, *Sipha flava* and one in Aleyrodidae, *Bemisia tabaci*, respectively. The species protein sequence was downloaded from the Insect base database [65]. To confirm the OBP, CSP, OR, IR, SNMP, GR genes families, we searched the protein sequences in the genomes of all 12 species using BLAST with the known genes from *A. pisum* as references, following the method used in the previous study [66]. We checked the conservative domains of the candidates manually in NCBI-CDD and removed these without the typical domain elements of the corresponding gene family. The reliable candidates were aligned using ClustalW software [60]. We constructed a phylogenetic tree using the neighbor-joining (NJ) method with the parameters of a Poisson model, complete deletion and 1000 bootstrap replicates, and visualized and improved the tree using the program Evolview (http://www.evolgenius.info/evolview/) [67].

### Expression profile of chemoreception genes

We extracted the total RNA from the whole body of the *S. chinensis* samples of three galls by the Trizol method [68], and then carried out library construction and Illumina HiSeq sequencing (2 × 150 bp) at Biomarker Technologies Corporation (Beijing, China). The obtained raw data underwent filtering, removal of adapters and primer sequences and elimination of low-quality sequences to obtain high-quality clean data by SeqPrep software (https://github.com/jstjohn/SeqPrep). Trinity software (https://github.com/trinityrnaseq/trinityrnaseq/wiki) was used to assemble the clean data [69]. Finally, the unigene sequences of *S. chinensis* were obtained. We used Blast software for a unigenes (> 150 bp) Blast search (e-value < 10^−5^ for all databases) and annotation against NR, Swiss-Prot, Pfam, COG, GO, and KEGG databases (e-value = 1 × 10^−6^) [70, 71]. We blasted the CDS sequences of the genomic chemoreception genes against unigene sequence of RNA database. The gene expression values are represented by transcript Fragments Per Kilobase of exon model per Million mapped fragments (FPKM). Genes with 100% similarity were identified as chemoreception genes expressed in the transcriptome of *S. chinensis.*

## Supplementary Information

**Additional file 1:** **Fig. S1.** Neighbor-joining tree of ORs of *S. chinensis* and other Hemiptera. Gene names are same as Fig. 6.

**Additional file 2:** **Fig. S2.** Neighbor-joining tree of GRs of *S. chinensis* and other Hemiptera. Gene names are same as Fig. 6.

**Additional file 3:** **Fig. S3.** Neighbor-joining tree of IRs of *S. chinensis* and other Hemiptera. Gene names are same as Fig. 6.

**Additional file 4:** **Fig. S4.** Neighbor-joining tree of SNMPs of *S. chinensis* and other Hemiptera. Gene names are same as Fig. 6.

**Additional file 5:** **Table S1.** Hi-C Assembly datastatistics of* Schlechtendalia chinensis.*

**Additional file 6:** **Table S2.** *Schlechtendalia chinensis *genome assembly detailed statistics.

**Additional file 7:** **Table S3.** The 10 conserved motifs of chemoreception genes family in the *Schlechtendalia chinensis.*

**Additional file 8:** **Table S4.** Nucleotide substitution rate of chemoreception gene in* Schlechtendalia chinensis.*

**Additional file 9:** **Table S5.** Number of reads generated from sequencing (clean data) and after quality filtering and adapter trimming (high quality data) for each sample.

**Additional file 10:** **Table S6.** The result of unigene Blast search and annotation of *Schlechtendalia chinensis.*

**Additional file 11:** **Table S7.** Evaluation of unigene/transcriptome Quality of *Schlechtendalia chinensis.*

## Data Availability

High-throughput sequencing data analyzed in this project and the whole-genome project (including assembly and annotation) are deposited under BioProject (PRJNA833747), BioSample (SAMN28016330) at NCBI GenBank. The whole-genome sequencing data are also available under Accession no. SRR23618925. The protein coding sequences of the other aphid species were downloaded from the Insect BASE website (http://v2.insect-genome.com/).

## References

[1] Bell J (1851). Chinese galls. Pharmaceut J.

[2] Tang C, Tsai PH (1957). Studies on the Chinese gallnuts of meitan Kweichow. Acta Entomol Sin.

[3] Zhang GX, Qiao GX, Zhong TS, Zhang WY. Fauna Sinica Insecta. Homoptera: Mindaridae and Pemphigidae. Science Press: Beijing, USA. 1999; p. 14.

[4] Baker AC (1917). On the Chinese gall (Aphididae-Hom). Ent News.

[5] Li ZG, Yang WY, Xia DJ (2003). Study on the Chinese gallnuts. For Res.

[6] Blackman RL, Eastop VF. Aphids on the World’s Crops: An Identification and Information Guide; John Wiley and Sons: New York, NY, USA, 1984.

[7] Heie OE (1980). The Aphidoidea (Hemiptera) of Fennoscandia and Denmark. I. general part, the families Mindaridae, Hor-maphididae, Thelaxidae, Anoeciidae, and Pemphigidae. Fauna. Entomol Scand.

[8] Remaudière G, Remaudière M (1997). Catalogue of the World’s Aphididae (Homoptera Aphidoidea).

[9] Ren  ZM, Su  X, Qiao  GX, von Dohlen CD ,  Wen  J (2018). *Nurudea zhengii Ren* and Qiao, a new species of the *Rhus* Gall Aphids (Aphididae: Eriosomatinae: Fordini) from Eastern China. Pakistan. J Zool.

[10] Zhang GX, Zhong TS. Economic Insect Fauna of China, Fasc. 25, Homoptera: Aphidinea; Science Press: Beijing China, 1983. (in Chinese).

[11] Yang ZX, Chen XM, Nathan H, Feng Y (2010). Phylogeny of *Rhus* gall aphids (Hemiptera: Pemphigidae) based on combined molecular analysis of nuclear EF1a and mitochondrial COII genes. Entomol Sci.

[12] Leal WS (2013). Odorant reception in insects: roles of receptors, binding proteins, and degrading enzymes. Annu Rev Entomol.

[13] Zhou JJ (2010). Odorant-binding proteins in insects. Vitam Horm.

[14] Xu YL, He P, Zhang L, Fang SQ, Dong SL, Zhang YJ (2009). Large-scale identification of odorant-binding proteins and chemosensory proteins from expressed sequence tags in insects. BMC Genomics.

[15] Vogt RG, Miller NE, Litvack R, Fandino RA, Sparks J, Staples J (2009). The insect SNMP gene family. Insect Biochem Mol Biol.

[16] Sun L, Wang Q, Wang Q, Dong K, Xiao Y, Zhang YJ (2017). Identification and characterization of odorant binding proteins in the forelegs of *Adelphocoris lineolatus* (Goeze). Front Physiol.

[17] Tang B, Tai S, Dai W, Zhang C (2019). Expression and functional analysis of two odorant-binding proteins from *Bradysia odoriphaga* (Diptera: Sciaridae). J Agric Food Chem.

[18] Chen  XF,  Xu  L, Zhang  YX, Wei  D, Wang  JJ,  Jiang  HB (2019). Genome-wide identification and expression profiling of odorant-binding proteins in the oriental fruit fly, *Bactrocera dorsalis*. Comp Biochem Physiol Part D Genomics Proteomics.

[19] Manoharan M, Chong  MNF,  Va¨ıtinadapoule  A, Frumence  ´E, Sowdhamini  R, Offmann  B (2013). Comparative genomics of odorant binding proteins in *Anopheles gambiae*, *Aedes aegypti*, and *Culex quinquefasciatus*. Genome Biol Evol.

[20] Vogt RG, Riddiford LM (1981). Pheromone binding and inactivation by moth antennae. Nature.

[21] Hekmat-Scafe DS, Scafe CR, McKinney AJ, Tanouye MA (2002). Genome-wide analysis of the odorant-binding protein gene family in *Drosophila melanogaster*. Genome Res.

[22] Waris MI, Younas A, Adeel MM, Duan SG, Quershi SR, Kaleem Ullah RM, et al. The role of chemosensory protein 10 in the detection of behaviorally active compounds in brown planthopper, *Nilaparvata lugens*. Insect Sci. 2018. 10.1111/1744-7917.12659 30593726

[23] Zeng Y, Merchant A, Wu Q, Wang S, Kong L, Zhou X, Xie W, Zhang Y (2020). A chemosensory protein BtabCSP11 mediates reproduction in *Bemisia tabaci*. Front Physiol.

[24] Kitabayashi AN, Arai T, Kubo T, Natori S (1998). Molecular cloning of cDNA for p10, a novel protein that increases in the regenerating legs of *Periplaneta americana* (American cockroach). Insect Biochem Mol Biol.

[25] Pikielny CW, Hasan G, Rouyer F, Rosbash M (1994). Members of a family of Drosophila putative odorant-binding proteins are expressed in different subsets of olfactory hairs. Neuron.

[26] Benton R, Sachse S, Michnick SW, Vosshall LB (2006). Atypical membrane topology and heteromeric function of Drosophila odorant receptors in vivo. PLoS Biol.

[27] Stengl  M, Funk  NW (2013). The role of the coreceptor Orco in insect olfactory transduction. J Comp Physiol A Neuroethol Sens Neural Behav Physiol.

[28] Brand P, Robertson HM, Lin W, Pothula R, Klingeman WE, Jurat-Fuentes JL, et al. The origin of the odorant receptor gene family in insects. Elife. 2018;7:e38340. 10.7554/eLife.38340 PMC6080948 30063003

[29] Saina M, Busengdal H, Sinigaglia C, Petrone L, Oliveri P, Rentzsch F, Benton R (2015). A cnidarian homologue of an insect gustatory receptor functions in developmental body patterning. Nat Commun.

[30] Clyne PJ, Warr CG, Freeman MR, Lessing D, Kim J, Carlson JR (1999). A novel family of divergent seven-transmembrane proteins: candidate odorant receptors in Drosophila. Neuron.

[31] Robertson HM (2019). Molecular evolution of the major arthropod chemoreceptor gene families. Annu Rev Entomol.

[32] Scott K (2018). Gustatory processing in *Drosophila melanogaster*. Annu Rev Entomol.

[33] Benton R, Vannice KS, Gomez-Diaz C, Vosshall LB (2009). Variant ionotropic glutamate receptors as chemosensory receptors in Drosophila. Cell.

[34] Rimal S, Lee Y (2018). The multidimensional ionotropic receptors of *Drosophila melanogaster*. Insect Mol Biol.

[35] Silbering AF, Rytz R, Grosjean Y, Abuin L, Ramdya P, Jefferis GS, Benton R (2011). Complementary function and integrated wiring of the evolutionarily distinct Drosophila olfactory subsystems. J Neurosci.

[36] Wu Z, Kang C, Qu M, Chen J, Chen M, Bin S, Lin J (2019). Candidates for chemosensory genes identified in the Chinese citrus fly, *Bactrocera minax*, through a transcriptomic analysis. BMC Genomics.

[37] Rogers  ME, Sun  M, Lerner  MR, Vogt  RG (1997). Snmp-1, a novel membrane protein of olfactory neurons of the silk moth Antheraea polyphemus with homology to the CD36 family of membrane proteins. J Biol Chem.

[38] Benton R, Vannice KS, Vosshall LB (2007). An essential role for a CD36-related receptor in pheromone detection in Drosophila. Nature.

[39] Ren Z, Zhu B, Wang D (2008). Comparative population structure of Chinese sumac aphid *Schlechtendalia chinensis*and its primary host-plant *Rhus* chinensis. Genetica.

[40] Lin R, Yang M, Yao B (2022). The phylogenetic and evolutionary analyses of detoxification gene families in Aphidinae species. PLoS ONE.

[41] Godfray HC (2010). The pea aphid genome. Insect Mol Biol.

[42] Quan Q, Hu X, Pan B, Zeng B, Wu N, Fang G (2019). Draft genome of the cotton aphid A*phis gossypii*. Insect Biochem Mol Biol.

[43] Schoonhoven LM, Van Loon A, Dicke M (2005). Insect-Plant Biology.

[44] Kawecki TJ (1998). Red queen meets Santa Rosalia: arms races and the evolution of host specialization in organisms with parasitic lifestyles. Am Nat.

[45] Pelosi P, Iovinella I, Felicioli A, Dani FR (2014). Soluble proteins of chemical communication: an overview across arthropods. Front Physiol.

[46] Vieira FG, Rozas J (2011). Comparative genomics of the odorant-binding and chemosensory protein gene families across the Arthropoda: origin and evolutionary history of the chemosensory system. Genome Biol Evol.

[47] Xin  Z, Huang  D, Zhao  D, Li  J, Wei  X, Xiao  J (2020). Genome-wide analysis of chemosensory protein genes (CSPs) family in fig wasps (Hymenoptera, Chalcidoidea). Genes (Basel).

[48] Kent LB, Robertson HM (2009). Evolution of the sugar receptors in insects. BMC Evol Biol.

[49] Andersson MN, Grosse-Wilde E, Keeling CI, Bengtsson JM, Yuen MMS, Li M, Hillbur Y, Bohlmann J, Hansson BS, Schlyter F (2013). Antennal transcriptome analysis of the chemosensory gene families in the tree killing bark beetles, *Ips typographus* and *Dendroctonus ponderosae* (Coleoptera: Curculionidae: Scolytinae). BMC Genomics.

[50] Cao J, Shi F (2012). Evolution of the RALF gene family in plants: Gene duplication and selection patterns. Evol Bioinf.

[51] Ma Y, Guo Z, Wang L (2022). The genome of the rice planthopper egg parasitoid wasps *Anagrus nilaparvatae* casts light on the chemo- and mechanosensation in parasitism. BMC Genomics.

[52] Forstner M, Gohl T, Gondesen I, Raming K, Breer H, Krieger J (2008). Differential expression of SNMP-1 and SNMP-2 proteins in pheromone-sensitive hairs of moths. Chem Senses.

[53] Jiang Q, Wang Z, Hu G, Yao X (2022). Genome-wide identification and characterization of AP2/ERF gene superfamily during flower development in *Actinidia eriantha*. BMC Genomics.

[54] Robertson HM, Wanner KW (2006). The chemoreceptor superfamily in the honey bee, *Apis mellifera*: expansion of the odorant, but not gustatory, receptor family. Genome Res.

[55] Kent CF, Minaei S, Harpur BA, Zayed A (2012). Recombination is associated with the evolution of genome structure and worker behavior in honey bees. Proc Natl Acad Sci U S A.

[56] Cannon SB, Mitra A, Baumgarten A, Young ND, May G (2004). The roles of segmental and tandem gene duplication in the evolution of large gene families in *Arabidopsis thaliana*. BMC Plant Biol.

[57] Tunstall NE, Sirey T, Newcomb RD, Warr CG (2007). Selective pressures on Drosophila chemosensory receptor genes. J Mol Evo.

[58] Robertson HM, Robertson ECN, Walden KKO, Enders LS, Miller NJ (2019). The chemoreceptors and odorant binding proteins of the soybean and pea aphids. Insect Bio chem Mol Biol.

[59] Geer LY, Geer RC, Gonzales NR (2011). CDD: A conserved domain database for the functional annotation of proteins. Nucleic Acids Res.

[60] Kumar S, Stecher G, Li M, Knyaz C, Tamura K (2018). MEGA X: molecular evolutionary genetics analysis across computing platforms. Mol Biol Evol.

[61] Bailey TL, Boden M, Buske FA, Frith M, Grant CE, Clementi L, Ren JY, Li WW, Noble WS (2009). MEME SUITE: Tools for motif discovery and searching. Nucleic Acids Res.

[62] Chen C, Chen H, Zhang Y, Thomas HR, Xia R (2020). TBtools: an integrative toolkit developed for interactive analyses of big biological data. Mol Plant.

[63] Chao  JT, Kong  YZ, Wang Q, Sun  YH, Gong  DP,  Lv  J, Liu  GS (2015). Mapgene2chrom, a tool to draw gene physical map based on perl and svg languages. Hereditas.

[64] Wang YP, Tang HB, DeBarry JD, Tan X, Li JP, Wang XY (2012). MCScanX: a toolkit for detection and evolutionary analysis of gene synteny and collinearity. Nucleic Acids Res.

[65] Yang M, Dong J, Shen YT, Xi C, Hao C (2022). Insect Base 2.0: a comprehensive gene resource for insects. Nucleic Acids Research.

[66] Zhou X (2015). Chemoreceptor evolution in Hymenoptera and its implications for the evolution of eusociality. Genome Biol Evol.

[67] Saitou N, Nei M (1987). The neighbor-joining method: A new method for reconstructing phylogenetic trees. Mol Biol Evol.

[68] Wang D (2012). An improved TRIzol method to extract total RNA from skin tissue of rana dybowskii. Chin J Wildl.

[69] Grabherr MG, Haas BJ, Yassour M (2011). Full length transcriptome assembly from RNA Seq data without a reference genome. Nat Biotechnol.

[70] Wang S, Yang Z, Pu Y (2016). De novo assembled transcriptome of horned gall aphid, *Schlechtendalia chinensis* Bell, suggest changes in functional gene expression during host alternation. Entomol&nbsp;Res.

[71] Kanehisa M, Furumichi M, Sato Y, Kawashima M, Ishiguro-Watanabe M (2023). KEGG for taxonomy-based analysis of pathways and genomes. Nucleic Acids Res.

