# Supplementary figures and images for: Genome-wide identification and characterization of the chemosensory relative protein genes in Rhus gall aphid Schlechtendalia chinensis
Source: BMC Genomics. 2023 Apr 28;24:222. doi: 10.1186/s12864-023-09322-4 (PMC10142413; doi:10.1186/s12864-023-09322-4)

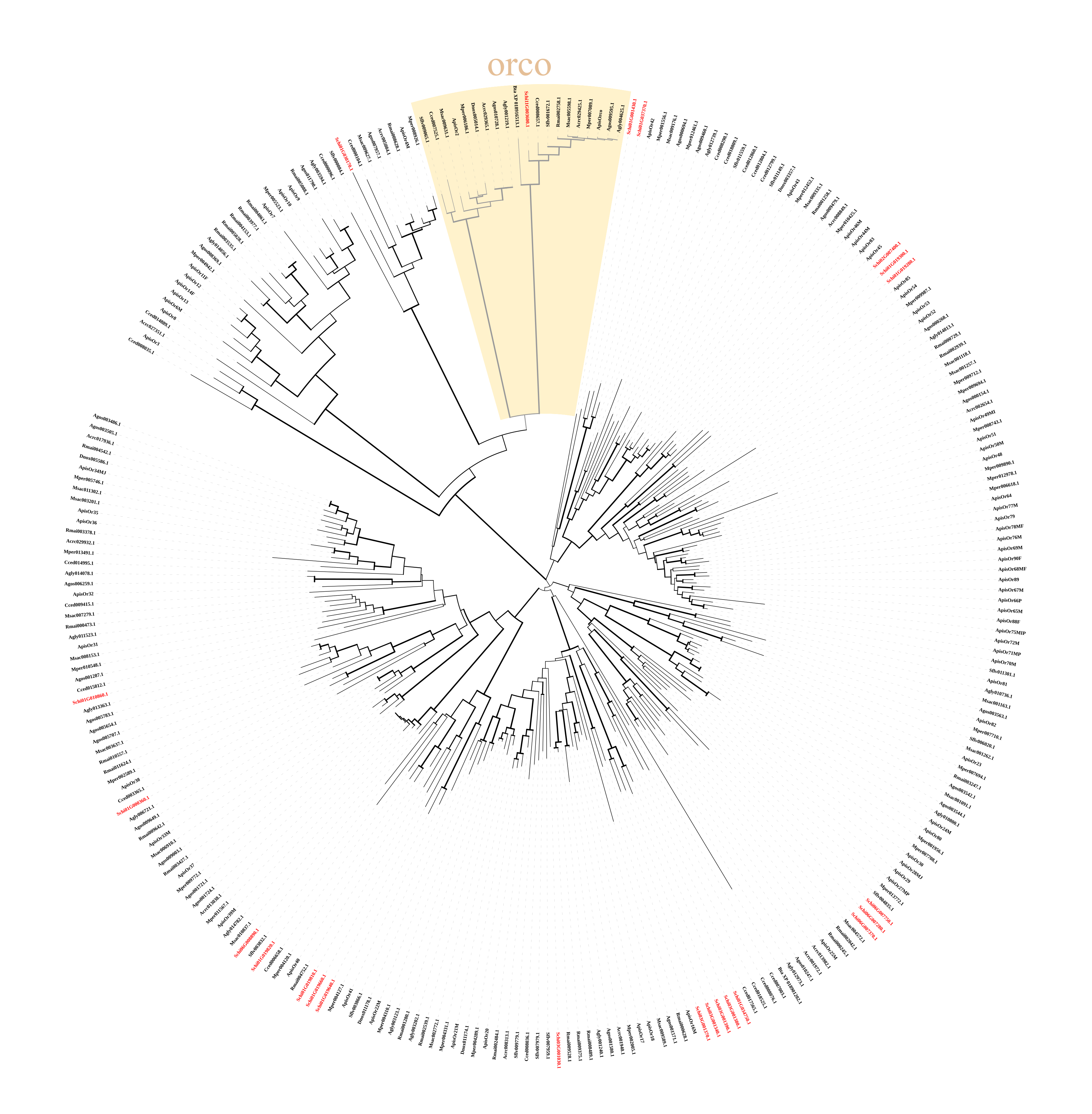

Supplement: Supplementary file 1 — Additional file 1: Fig. S1. Neighbor-joining tree of ORs of S. chinensis and other Hemiptera. Gene names are same as Fig. 6. [file 12864_2023_9322_MOESM1_ESM.png]

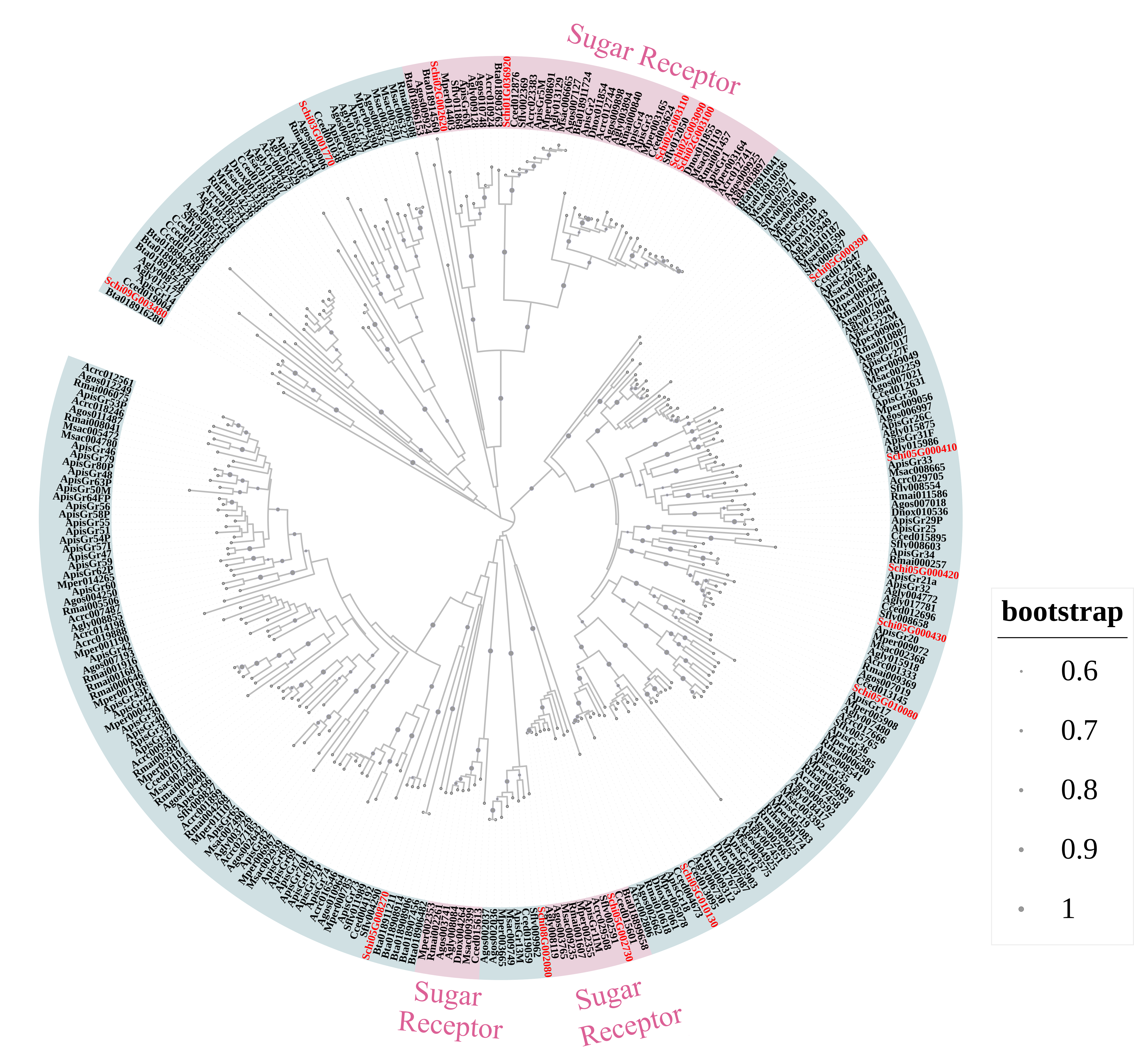

Supplement: Supplementary file 2 — Additional file 2: Fig. S2. Neighbor-joining tree of GRs of S. chinensis and other Hemiptera. Gene names are same as Fig. 6. [file 12864_2023_9322_MOESM2_ESM.png]

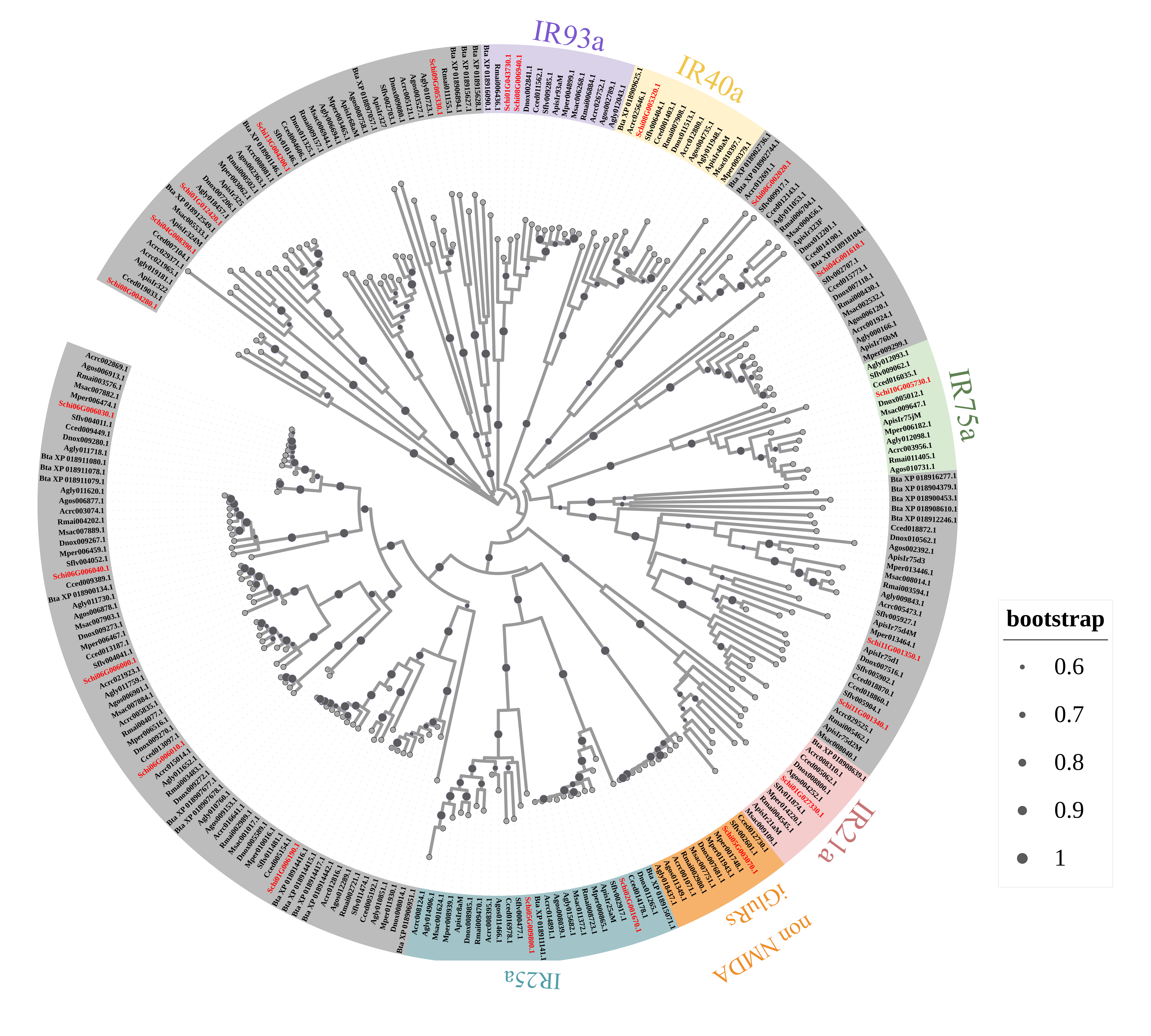

Supplement: Supplementary file 3 — Additional file 3: Fig. S3. Neighbor-joining tree of IRs of S. chinensis and other Hemiptera. Gene names are same as Fig. 6. [file 12864_2023_9322_MOESM3_ESM.png]

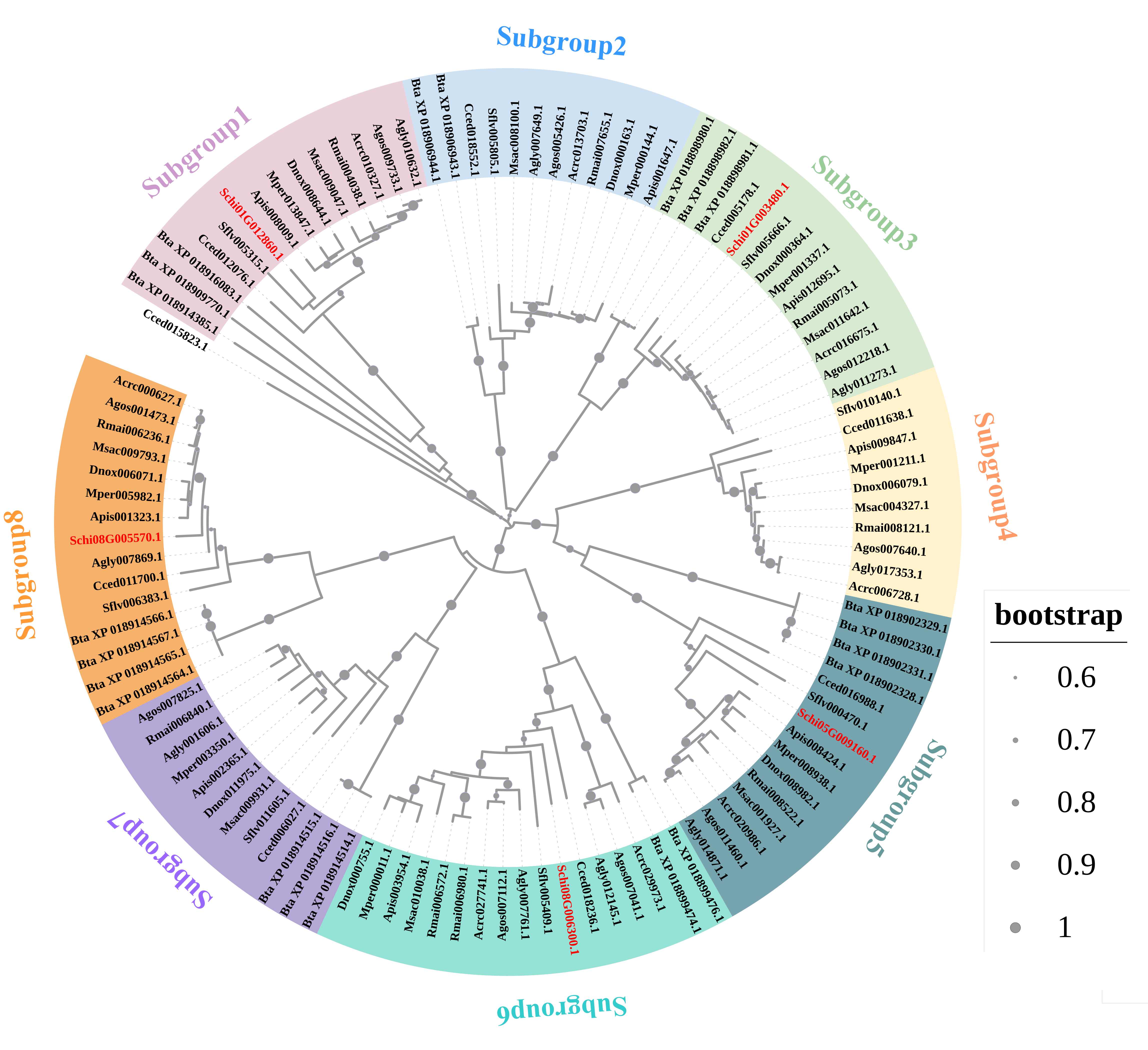

Supplement: Supplementary file 4 — Additional file 4: Fig. S4. Neighbor-joining tree of SNMPs of S. chinensis and other Hemiptera. Gene names are same as Fig. 6. [file 12864_2023_9322_MOESM4_ESM.png]
