# Supplementary material for: Genome-wide identification and characterization of the chemosensory relative protein genes in Rhus gall aphid Schlechtendalia chinensis
Source: BMC Genomics. 2023 Apr 28;24:222. doi: 10.1186/s12864-023-09322-4 (PMC10142413; doi:10.1186/s12864-023-09322-4)
Supplement: Supplementary file 5 — Additional file 5: Table S1. Hi-C Assembly datastatistics of Schlechtendalia chinensis. [file 12864_2023_9322_MOESM5_ESM.docx]

**Supporting information for**

**Genome-Wide Identification and Characterization of the Chemosensory Relative Protein Genes in *Rhus* Gall Aphid *Schlechtendalia chinensis***

**Hongli He ^1^, M. James C. Crabbe ^1,2,3^ and Zhumei Ren ^1,*^**

^1^School of Life Science, Shanxi University, Taiyuan, Shanxi 030006, China;

^2^Wolfson College, Oxford University, Oxford OX2 6UD, UK

^3^Institute of Biomedical and Environmental Science & Technology, University of Bedfordshire, Luton LU1 3JU, UK

*Correspondence: zmren@sxu.edu.cn

**Table S1. Hi-C Assembly data statistics of *Schlechtendalia chinensis***

| Group | Cluster Num | Cluster Len | Oder Num | Oder Len |
| --- | --- | --- | --- | --- |
| LG01 | 11 | 123,018,421 | 8 | 122,785,658 |
| LG02 | 1 | 22,033,345 | 1 | 22,033,345 |
| LG03 | 1 | 21,282,667 | 1 | 21,282,667 |
| LG04 | 1 | 21,091,861 | 1 | 21,091,861 |
| LG05 | 2 | 20,798,509 | 1 | 20,977,384 |
| LG06 | 1 | 20,798,509 | 1 | 20,798,509 |
| LG07 | 1 | 15,397,934 | 1 | 15,397,934 |
| LG08 | 1 | 13,329,565 | 1 | 13,329,565 |
| LG09 | 5 | 12,370,492 | 5 | 12,217,479 |
| LG10 | 2 | 12,226,705 | 1 | 12,191,903 |
| LG11 | 1 | 11,734,047 | 1 | 11,734,047 |
| LG12 | 1 | 11,250,006 | 1 | 11,250,006 |
| LG13 | 1 | 10,465,550 | 1 | 10,465,550 |
| Total(Ratio) | 29(14.8) | 315996691(91.71) | 20(68.97) | 315555908(99.86) |
