# Supplementary material for: Genome-wide identification and characterization of the chemosensory relative protein genes in Rhus gall aphid Schlechtendalia chinensis
Source: BMC Genomics. 2023 Apr 28;24:222. doi: 10.1186/s12864-023-09322-4 (PMC10142413; doi:10.1186/s12864-023-09322-4)
Supplement: Supplementary file 6 — Additional file 6: Table S2. Schlechtendalia chinensis genome assembly detailed statistics. [file 12864_2023_9322_MOESM6_ESM.docx]

**Table S2.** ***Schlechtendalia chinensis* genome assembly detailed statistics**

| Assembly features | Hi-C Scaffolds |
| --- | --- |
| Number of chromosomes (n) | 13 |
| Number of Scaffolds | 189 |
| Total size of Scaffolds | 344.56 Mb |
| Longest scaffold size (Mbp) | 122.78 Mb |
| Shortest Scaffold size (Mbp) | 15.65 Kb |
| Mean scaffold length (Mbp) | 1.82 Mb |
| Median scaffold size | 77.06 Kb |
| N50 scaffold length | 21.09 Mb |
| L50 scaffold count | 10 |
| Scaffolds GC content | 33.74% |
| Scaffolds Gaps (N) content | 0 |
| Percentage of assembled contigs in scaffolds | 96.92% |
| Average number of contigs per scaffold | 1.03 |
| BUSCO (complete) | 94.34% |
| **Gene models** |  |
| Number of genes models | 15,289 |
| Mean coding sequence length CDS | 1520.26 bp |
| Mean number of exons per gene | 6.61 |
| Mean exon length | 1822.17 bp |
| Mean intron length | 6663.72 bp |
| Non-protein-coding genes | 426 |
| Number of miRNA gene | 23 |
| Number of tRNA gene | 136 |
| Number of rRNA gene | 33 |
| Number of snRNA gene | 71 |
| **Pseudogene** |  |
| Number of pseudogenes | 192 |
| Total length | 523,288 |
| Average length | 2725.46 |
