# Supplementary material for: Genome-wide identification and characterization of the chemosensory relative protein genes in Rhus gall aphid Schlechtendalia chinensis
Source: BMC Genomics. 2023 Apr 28;24:222. doi: 10.1186/s12864-023-09322-4 (PMC10142413; doi:10.1186/s12864-023-09322-4)
Supplement: Supplementary file 7 — Additional file 7: Table S3. The 10 conserved motifs of chemoreception genes family in the Schlechtendalia chinensis. [file 12864_2023_9322_MOESM7_ESM.docx]

**Table S3.** **The 10 conserved motifs of chemoreception genes family in the *Schlechtendalia chinensis***

| Gene  Family | Motif | Length (aa) | Amino acid conserved sequence |
| --- | --- | --- | --- |
| OBPs | Motif1 | 29 | PNDRNYKCFLKCGFEEVGLIDDQGIIDGE |
|  | Motif2 | 15 | GRPDGCETAYNFMVC |
|  | Motif3 | 50 | TLSSSSFVFGILVVMLVGQMTARPQSAEEETMKKELYKVCSGKYPITEEM |
|  | Motif4 | 41 | LMRLYNEGVQDRNYYMATLSAVRHCISIAQQLKQLQPSKKF |
|  | Motif5 | 15 | WKSTWKKSLDKCFDD |
|  | Motif6 | 21 | YYGTNQTLMKKAKDLIDVCAK |
|  | Motif7 | 29 | WLAYESNCPNDKIKHTKKCEKTRKRFRMQ |
|  | Motif8 | 39 | DTADKELMSKLFTVVFKCFKDADWGTCGEMITTKYDITQ |
|  | Motif9 | 29 | DRSATIFQSCIAETKLSGDALKGFRSMSI |
|  | Motif10 | 29 | MRGNYFATFFLLFGFGLQEISCQTHELSG |
| ORs | Motif1 | 50 | QMYMYCYIFDTLHNEKDSIIFGLYSCNWTEMDMKCKKLILLTMRMNNAHQ |
|  | Motif2 | 21 | KVSPKSIVNLEMFARVMNMSY |
|  | Motif3 | 21 | DHQNVYLKMKSFYAVVRPITL |
|  | Motif4 | 19 | INLKMLKQFGFYQMFDPNS |
|  | Motif5 | 29 | DRLRGVLDAARYRYTAAGRADPSRLRRCR |
|  | Motif6 | 17 | ALSAQFRTVAAAYETLG |
|  | Motif7 | 15 | TVHNWWYPVSEAAFN |
|  | Motif8 | 21 | ADQIWELFDLARLDFLTSKRC |
|  | Motif9 | 21 | QIFVSSLSVIMLWFIFIMSFS |
|  | Motif10 | 27 | ASLSVWLRAFVAYHAVATAVWTAAPWF |
| GRs | Motif1 | 29 | TACDFFTLNTHLITSAIAAGTTYLVILLQ |
|  | Motif2 | 29 | TDINNRYLDNNTKEELQLFLNQISSCTIE |
|  | Motif3 | 50 | PEEWTRSITGMTLDKIRLLHAELSDLLRLFSMGFGQILLGFFVFSYIDML |
|  | Motif4 | 50 | MANAVEVLRIKHWLTRQAVDILNNLLGIQMGLSVFLLWVMALFDIYYEIF |
|  | Motif5 | 41 | YQLFMYVQNLSMCCIETQFVVQCFKVYTKFRGINDDLKRLK |
|  | Motif6 | 39 | SPSKILVYGWLLQYTSRLFMIVLVAHYTTKQAMKSKSLI |
|  | Motif7 | 39 | NFVVITKYFFGIEYVLDVAVCFSTYFFLQQLEYRFQTLN |
|  | Motif8 | 42 | MNGIIEYDQKLTSLPRFLLIRQHLPKKSYWNIILIFTLIFYI |
|  | Motif9 | 50 | WNYMDLFIIILSCALSDKFKQLNQKLATVKGKVLPSTYWRKSRETYNLLA |
|  | Motif10 | 29 | PHIVYLKNVVLVLAIIIAASRVHEKKRKM |
| IRs | Motif1 | 41 | FFMESSSIEYEVQRNCDLTEVGYWLDNKAYGIAMPFNAPHR |
|  | Motif2 | 17 | TLIMVSSYTANLAAFLT |
|  | Motif3 | 31 | NKWNGIIGEVQEMRADLGVCDLTITHERRSA |
|  | Motif4 | 21 | QQGSDMAPKAVSTRIVAGMWW |
|  | Motif5 | 29 | MATAYLGVSVLLYILARFSPYEWENPHPC |
|  | Motif6 | 32 | KGYCIDLLVGLSKDINFTYSLSLSPDGQFGNY |
|  | Motif7 | 21 | GGVFVVLMGGMGVACVVAVFE |
|  | Motif8 | 21 | KKPSRSSTLVSFLQPFSHTLW |
|  | Motif9 | 29 | SLAILDLQEKGEIQMLYNKWWKPPNDMCA |
|  | Motif10 | 38 | DKQSAIKYGCVRGQSTASFFENSDVNLYQKMWSVMETY |
| CSPs | Motif1 | 29 | RILTSYIKCLLDEGNCTNEGRELRKVLPD |
|  | Motif2 | 41 | AIQTECAKCSEQQRRQAGKVLAHLLTYKPEYWKMLVQKFDP |
|  | Motif3 | 21 | AEEKYTTKFDNFDVDKVLNNH |
|  | Motif4 | 33 | RSSSSVTMKVFVIAVCVCAALARPEDSKVENKP |
|  | Motif5 | 21 | QHDSSIPNVSEDILDKALGDR |
| SNMPs | Motif1 | 21 | EMWKTPEAAVYLKVYIFNVTN |
|  | Motif2 | 21 | EKPKLQEIGPFVYRENMEKVN |
|  | Motif3 | 21 | CYYGFPIALSYPHFYKSDRSL |
|  | Motif4 | 25 | LDDVEGLSPDQEKHETFLKIQPRLG |
|  | Motif5 | 21 | GQVIRFNGKTKMNVWPGDECN |
|  | Motif6 | 29 | IHFNNNGTVTYQHKKILEFMPHLSVISND |
|  | Motif7 | 21 | DLVHVYDKDLCRVWALRYRKD |
|  | Motif8 | 22 | PFVRQTAKEFMFGYESPLVTIG |
|  | Motif9 | 9 | NPDNACFCE |
|  | Motif10 | 49 | RENIERGRQSILRRGSSMLVNGQHRLLIVRESYHRISHTMQETDVDAQQ |
