# Supplementary material for: Genome-wide identification and characterization of the chemosensory relative protein genes in Rhus gall aphid Schlechtendalia chinensis
Source: BMC Genomics. 2023 Apr 28;24:222. doi: 10.1186/s12864-023-09322-4 (PMC10142413; doi:10.1186/s12864-023-09322-4)
Supplement: Supplementary file 8 — Additional file 8: Table S4. Nucleotide substitution rate of chemoreception gene in Schlechtendalia chinensis. [file 12864_2023_9322_MOESM8_ESM.docx]

**Table S4. Nucleotide substitution rate of chemoreception gene in** ***Schlechtendalia chinensis***

| Chromosome | Gene family | Gene ID | Chromosome | Gene ID | Ka | ks | Ka/Ks |
| --- | --- | --- | --- | --- | --- | --- | --- |
| LG01 | SNMP | Schi01G012860 | ChrA1 | Apis008009 | 0.1405 | 1.1302 | 0.1243 |
| LG01 | OR | Schi01G019810 | ChrA1 | Apis006180 | 0.2884 | 0.6514 | 0.4427 |
| LG01 | OBP | Schi01G026560 | ChrA1 | Apis006783 | 0.4489 | 1.2635 | 0.3553 |
| LG01 | CSP | Schi01G030200 | ChrA3 | Apis015305 | 0.3489 | 1.4418 | 0.2420 |
| LG01 | GR | Schi01G036920 | ChrA1 | Apis008097 | 0.0886 | 1.0558 | 0.0839 |
| LG02 | GR | Schi02G002620 | ChrA3 | Apis013251 | 0.1251 | 1.5183 | 0.0824 |
| LG03 | OR | Schi03G001030 | ChrA3 | Apis012846 | 0.3990 | 0.8859 | 0.4503 |
| LG03 | OR | Schi03G001210 | ChrA3 | Apis011327 | 0.1022 | 1.4885 | 0.0686 |
| LG03 | OBP | Schi03G001280 | ChrA3 | Apis010883 | 0.2358 | 1.8650 | 0.1264 |
| LG03 | CSP | Schi03G005120 | ChrA3 | Apis014398 | 1.0547 | 1.4148 | 0.7455 |
| LG04 | IR | Schi04G001610 | ChrA2 | Apis010137 | 0.0974 | 0.7258 | 0.1342 |
| LG04 | OBP | Schi04G002700 | ChrA2 | Apis010484 | 0.2280 | 1.5505 | 0.1471 |
| LG04 | OBP | Schi04G002720 | ChrA2 | Apis009154 | 0.1328 | 0.8768 | 0.1515 |
| LG05 | GR | Schi05G000390 | ChrA1 | Apis006094 | 0.6317 | 1.7960 | 0.3517 |
| LG05 | GR | Schi05G002730 | ChrA3 | Apis012065 | 0.9447 | 1.6377 | 0.5768 |
| LG05 | IR | Schi05G003070 | ChrA3 | Apis010777 | 0.0141 | 0.8730 | 0.0161 |
| LG05 | IR | Schi05G009800 | ChrA1 | Apis008348 | 0.1042 | 1.3066 | 0.0797 |
| LG06 | OR | Schi06G007750 | ChrA1 | Apis008496 | 0.4566 | 1.4157 | 0.3226 |
| LG08 | GR | Schi08G002080 | ChrX | Apis003098 | 0.1876 | 0.6664 | 0.2816 |
| LG08 | IR | Schi08G005320 | ChrX | Apis002055 | 0.1404 | 1.1626 | 0.1208 |
| LG08 | SNMP | Schi08G005570 | ChrX | Apis001323 | 0.0589 | 1.3460 | 0.0437 |
| LG08 | SNMP | Schi08G006300 | ChrX | Apis003954 | 0.1334 | 1.2029 | 0.1109 |
| LG08 | IR | Schi08G006940 | ChrX | Apis001472 | 0.0931 | 1.0395 | 0.0895 |
| LG09 | CSP | Schi09G001230 | ChrA3 | Apis012074 | 0.5926 | 1.5987 | 0.3706 |
| LG13 | OBP | Schi13G001840 | ChrA1 | Apis005023 | 0.1198 | 1.2955 | 0.0925 |
| LG13 | IR | Schi13G004200 | ChrA2 | Apis009471 | 0.1136 | 0.6275 | 0.1811 |
