# Supplementary material for: Genome-wide identification and characterization of the chemosensory relative protein genes in Rhus gall aphid Schlechtendalia chinensis
Source: BMC Genomics. 2023 Apr 28;24:222. doi: 10.1186/s12864-023-09322-4 (PMC10142413; doi:10.1186/s12864-023-09322-4)
Supplement: Supplementary file 9 — Additional file 9: Table S5. Number of reads generated from sequencing (clean data) and after quality filtering and adapter trimming (high quality data) for each sample. [file 12864_2023_9322_MOESM9_ESM.docx]

**Table S5. Number of reads generated from sequencing (clean data) and after quality filtering and adapter trimming (high quality data) for each sample**

| Sample name | Clean read | Clean bases | Errow rate（%） | Q20% | Q30% | GC% |
| --- | --- | --- | --- | --- | --- | --- |
| Ren_IA4601 | 41,891,256 | 6,259,599,115 | 0.0288 | 96.47 | 90.79 | 47.67 |
| Ren_IA4603 | 42,713,770 | 6,382,150,904 | 0.0277 | 96.95 | 91.73 | 42.82 |
| Ren_IA4621 | 46,481,158 | 6,924,341,276 | 0.0283 | 96.68 | 91.28 | 48.39 |
