# Supplementary material for: Genome-wide identification and characterization of the chemosensory relative protein genes in Rhus gall aphid Schlechtendalia chinensis
Source: BMC Genomics. 2023 Apr 28;24:222. doi: 10.1186/s12864-023-09322-4 (PMC10142413; doi:10.1186/s12864-023-09322-4)
Supplement: Supplementary file 10 — Additional file 10: Table S6. The result of unigene Blast search and annotation of Schlechtendalia chinensis. [file 12864_2023_9322_MOESM10_ESM.docx]

**Table S6. The result of unigene Blast search and annotation of *Schlechtendalia chinensis***

|  | Exp_Unigene number(percent) | Exp_Transcript number(percent) | All_Unigene number(percent) | All_Transcript number(percent)GO |
| --- | --- | --- | --- | --- |
| KEGG | 6409(0.2621) | 12023(0.3102) | 6431(0.2565) | 12150(0.3061) |
| COG | 10496(0.4292) | 19474(0.5024) | 10551(0.4207) | 19682(0.4958) |
| NR | 11585(0.4737) | 21468(0.5538) | 11653(0.4647) | 21705(0.5468) |
| Swiss-Prot | 8107(0.3315) | 15168(0.3913) | 8135(0.3244) | 15322(0.386) |
| Pfam | 8880(0.3631) | 16419(0.4236) | 8912(0.3554) | 16579(0.4176) |
| Total_anno | 11876(0.4856) | 21862(0.564) | 11953(0.4767) | 22111(0.557) |
| Total | 24454(1.0) | 38764(1.0) | 25077(1.0) | 39698(1.0) |
