# Supplementary material for: Genome-wide identification and characterization of the chemosensory relative protein genes in Rhus gall aphid Schlechtendalia chinensis
Source: BMC Genomics. 2023 Apr 28;24:222. doi: 10.1186/s12864-023-09322-4 (PMC10142413; doi:10.1186/s12864-023-09322-4)
Supplement: Supplementary file 11 — Additional file 11: Table S7. Evaluation of unigene/transcriptome Quality of Schlechtendalia chinensis. [file 12864_2023_9322_MOESM11_ESM.docx]

**Table S7. Evaluation of unigene/transcriptome Quality of *Schlechtendalia chinensis***

| Type | unigene | transcript |
| --- | --- | --- |
| Total number | 25077 | 39698 |
| Total base | 30032523 | 54628181 |
| Largest length (bp) | 27571 | 27571 |
| Smallest length (bp) | 201 | 201 |
| Average length (bp) | 1197.61 | 1376.09 |
| N50 length (bp) | 2175 | 2261 |
| E90N50 length (bp) | 2890 | 2713 |
| Mean mapped percent (%) | 81.02 | 86.171 |
| GC percent (%) | 40.08 | 41.39 |
| Trans Rate score | 0.27281 | 0.32261 |
| BUSCO score | 89.3% (3.1%) | 89.3% (3.1%) |
